# Read-Across Structural Analysis of PFAS Acute Oral Toxicity in Rats Powered by the Isalos Analytics Platform’s Automated Machine Learning

**DOI:** 10.3390/toxics14020152

**Published:** 2026-02-03

**Authors:** Aikaterini Theodori, Konstantinos D. Papavasileiou, Andreas Tsoumanis, Georgia Melagraki, Antreas Afantitis

**Affiliations:** 1 Department of ChemInformatics, NovaMechanics MIKE, 18545 Piraeus, Greece; theodori@novamechanics.com (A.T.); papavasileiou@novamechanics.com (K.D.P.); tsoumanis@novamechanics.com (A.T.); 2 Department of ChemInformatics, NovaMechanics Ltd., Nicosia 1070, Cyprus; 3 Entelos Institute, Larnaca 6059, Cyprus; 4 Division of Physical Sciences Applications, Hellenic Military Academy, 16672 Vari, Greece; georgiamelagraki@gmail.com; 5 Department of Pharmacy, Frederick University, Nicosia 1036, Cyprus

**Keywords:** per- and polyfluoroalkyl substances, PFAS, acute oral toxicity, LD50, rats, machine learning, read-across, structural analysis, Enalos Cloud Platform

## Abstract

The ubiquity and environmental persistence of per- and polyfluoroalkyl substances (PFASs) have raised significant concerns about their detrimental effects on human health. Collective scientific efforts are increasingly focused on elucidating PFAS toxicity mechanisms and identifying potential low-impact PFAS structures that retain the exceptional properties of this chemical class. To advance the use of in silico methods in PFAS toxicity assessment, we developed a robust modelling framework for predicting PFAS acute oral toxicity class (high or low) in rats, leveraging the enhanced capabilities of the in-house Isalos Analytics Platform. The automated machine learning (autoML) functionality was employed to optimise four ML models—k-nearest neighbours (kNN), Random Forest (RF), eXtreme Gradient Boosting (XGBoost), and fully connected neural network (NN)—using Mold2 molecular descriptors, and to identify the top-performing model through five-fold cross-validation. The selected kNN model (k = 3) was used for predictions on the held-out testing set, achieving an accuracy of 81.5%, while a Shapley values analysis provided valuable insights into the factors influencing toxicity predictions. Furthermore, the nearest-neighbour-based methodology enabled a read-across structural analysis of PFAS similarity groups consisting of each testing set instance and its three closest neighbours in the training set. This analysis revealed a consistent association between polyaromatic and heterocyclic structural features and high acute oral toxicity. The developed, thoroughly validated read-across model is freely accessible through the INSIGHT RatTox web application as well as the INSIGHT Cheminformatics Platform in Enalos Cloud, supporting high-throughput screening of PFAS compounds and investigation of structural similarities with their nearest neighbours for enriched structural interpretation.

## 1. Introduction

The relationship between humans and the environment has been central to survival and evolution, shaping the traditional, cultural, and societal development of both early and modern civilizations [1]. The reciprocity of this relationship is grounded in maintaining a balance between human demands and environmental stewardship; however, in the past few decades, the rapid and largely unregulated growth of industrial and technological activities has exerted considerable pressure on environmental systems. In turn, environmental degradation and pollution have begun to manifest clear and accelerating adverse effects on living organisms and human health [2,3]. As a response to these emerging challenges, the World Health Organization (WHO) has proposed a global strategy aimed at the prevention, communication, and mitigation of environmentally related threats to human health and well-being by 2030. Among the primary goals of this initiative is to manage and reduce chemical pollution in soil, air, and waters [4].

A key contributor to the aforementioned issues is a group of human-made, persistent chemicals: the class of per- and polyfluoroalkyl substances (PFASs), also known as “forever chemicals”. These organofluorine compounds have found industrial and commercial applications since their production in the early 1950s [5]; however, their impact on human health was only studied decades later. Analysis of archived human plasma samples has revealed the presence of PFASs in human blood since at least the 1980s [6]. Consequently, growing concern over the implications of these versatile chemicals has prompted multiple attempts towards the creation of a representative classification and identification framework, which could support the development of universal restriction protocols [7]. Yet, despite these efforts, as many as nine distinct PFAS definitions are currently in use [8]. The most recent PFAS definition by the Organisation for Economic Co-operation and Development (OECD) was revised in 2021 to encompass the broadest range of fluorinated compounds to date, stating that, with few exceptions, “PFAS include every substance that contains at least one fully fluorinated methyl (–CF_3_) or methylene (—CF_2_—) carbon atom” [9]. Within this continuously expanding category of chemicals, recent database searches have identified up to 7 million potential PFAS structures [10]. To address the complexity and diversity of this rapidly growing chemical space, recent advances in computational modelling have emerged, including SimpleBox4Planet, a multimedia fate model developed by our group and implemented in the Enalos Cloud Platform to enable environmental fate and exposure simulations for thousands of PFAS structures across diverse scenarios [11].

PFAS applications are enabled by their unique chemical and structural properties, which render them invaluable in a wide range of industrial processes as well as in everyday consumer products. Their surfactant properties, for example, are exploited in the mining and oil industries to facilitate metal and oil extraction, while their exceptional water- and oil-repellent characteristics make them valuable as coating agents in various material-processing applications. Moreover, their mechanical and physicochemical stability has been utilised in the manufacture of novel plastic materials, as well as in processes for metal and solvent recovery [12]. Consumer applications include, but are not limited to, coatings, paints and varnishes, medical equipment, packaging materials, and cosmetic products. As a result, PFAS exposure pathways arise from the use of everyday items—such as non-stick cookware, contact lenses, and food packaging—but also from environmental releases associated with industrial activities, wastewater treatment, and solid-waste management [13,14].

The multiple pathways through which PFAS enter the human body have contributed to their bioaccumulation, intensifying concerns about their potential toxicity and driving increased scientific investigation into their detrimental effects. In vitro studies have shown potential associations between PFAS exposure and elevated triglyceride levels in human liver cells [15], adverse effects on the immune system [16], as well as reactive oxygen species (ROS)-induced autophagy and apoptosis in human embryonic liver cells [17]. Additionally, bioaccumulation of perfluorooctane sulfonate (PFOS) in the liver, lungs, kidneys, spleen, heart, and brain of laboratory mice has been linked to liver and pericardial damage as well as neurotoxic effects [18], whereas exposure to perfluorohexane sulfonate (PFHxS) has been shown to cause thyroid hormone dysregulation in rats [19]. Human studies remain limited; however, there is evidence suggesting PFAS toxicity. For instance, exposure to PFOS and perfluorooctanoic acid (PFOA) has been associated with disruptions in oestrogen levels during pregnancy and foetal growth restrictions [20].

In response to the potential threats to human health, strict regulations are in place to limit PFAS use and human exposure. Most recently, the European Union (EU) adopted a new restriction that will prohibit the use of PFAS in firefighting foams containing more than 1 mg/L of total PFAS [21], while the United States (U.S.) Environmental Protection Agency (EPA) has implemented the first federal drinking-water standard for PFASs, establishing legally enforceable limits for several compounds in public water systems [22]. Evidence from previous regulatory initiatives targeting PFAS use shows a clear decline in legacy PFAS concentrations (e.g., PFOS and PFOA) in human blood from the early 2000s to 2021 across Danish, Australian, and U.S. populations [23,24], yet data on emerging PFAS compounds are limited. As a further step, alternative chemicals are being investigated to replace PFAS due to their associated adverse toxic effects; however, their exceptional properties render them indispensable in numerous applications [25].

While progress is being made toward the phase-out of PFASs, it is clear that the goal is not yet attainable. Nonetheless, alternative approaches have been increasingly explored, with growing efforts to leverage advancements in data analysis and machine learning (ML) to elucidate PFAS toxicity mechanisms [26]. The resulting knowledge can then be used to establish “rules” of PFAS toxicity, identify low-toxicity structures that retain desirable functional properties, and ultimately evaluate their potential as alternative, low-impact PFAS for critical applications. For example, Hu et al. combined ML, molecular dynamics, and experimental approaches to accurately predict key biodistribution and bioaccumulation parameters, as well as toxicity factors [27].

Purely ML-based studies have also demonstrated strong predictive performance, as illustrated, for instance, by quantitative structure-activity relationship (QSAR) models developed for PFAS bioactivity classification [28]. Complementing these approaches, in-house read-across and consensus models for PFAS cytotoxicity and binding affinity prediction to nuclear receptors (e.g., PPARγ and PPARδ) further demonstrate the utility of in silico methods for toxicity assessment [29,30]. Beyond such bioactivity- and mechanism- oriented models, numerous in silico studies focus on the prediction of the most common toxicity endpoints, acute oral (LD_50_) and acute inhalation (LC_50_) toxicity, which are measured as the dose that produces 50% mortality in the tested population following oral or inhalation exposure [31,32,33,34,35,36]. Accordingly, a range of downloadable and web-based in silico tools has been developed to support acute toxicity predictions. Among these, the Collaborative Acute Toxicity Modelling Suite (CATMoS) provides advanced chemical toxicity predictions based on hundreds of pre-trained models derived from a broad chemical database [37]. Similarly, the Toxicity Estimation Software Tool (TEST, version 5.1.2), available through the EPA’s CompTox Chemicals Dashboard, employs linear QSAR models to estimate acute oral toxicity as a function of selected molecular descriptors [38].

Building on the synergy between ML and structural analysis of chemical compounds, read-across methods provide advanced and interpretable predictions by grouping chemicals based on structural similarity and using the attribute values of the most similar structure to infer those of unknown compounds [39,40]. Naturally, the accuracy and reliability of read-across models depend both on the confidence of the model evaluation and, importantly, on the uncertainty associated with the similarity criteria used for grouping [41]. A successful example of the application of read-across methodologies for toxicity prediction is the toxFlow web application, which predicts nanoparticle toxicity using physicochemical and biological criteria for similarity assessment [42]. Of particular relevance to chemical toxicity evaluation, rat acute oral toxicity predictions have been enabled through read-across models that rely on hybrid chemical and bioprofiling similarity criteria [43], as well as similarity measures derived from data-driven analyses [44]. Additionally, the ProTox web server supports similarity-based rodent LD_50_ predictions and provides associated toxic fragment and potential toxicity targets identification for a wide range of chemicals [45].

In the effort toward PFAS use regulation, toxicity assessment, and, eventually, their replacement with safer analogues in key industrial and commercial applications, the lack of experimental data and challenges associated with interpreting ML model outputs pose serious impediments. In the present work, we aim to develop a highly accurate model for predicting PFAS acute oral toxicity, capable of classifying existing or emerging compounds into high- or low-toxicity categories. Such classification can support the rapid screening of diverse PFAS structures and facilitate the identification of low-toxicity candidates. To enhance our predictive results and deepen our understanding of PFAS toxicity mechanisms, a central goal of this study is the application of ML-based read-across methodologies, which provide valuable insight into the model’s decision process and the structural similarities that may underlie toxic effects. Data on PFAS acute oral toxicity in rats were compiled from studies published by Chen et al. [46], da Silva and de Melo [47], and Lu et al. [48], and enhanced with calculated molecular descriptors. The automated ML option (AutoML) in Isalos Analytics Platform was used to optimise four ML methods—k-nearest neighbours (kNN) [49], random forest (RF) [50], eXtreme Gradient Boosting (XGBoost) [51], and a fully connected neural network (NN). The top-performing model in the internal validation tests was the neighbour-based kNN model, which was subsequently used for testing set predictions and Shapley value analysis. Importantly, it also enabled structural analysis of toxicity patterns in PFAS molecules by treating the k nearest neighbours identified for each new observation as similarity groups. The final model was made available as the INSIGHT RatTox web service as well as a component of the INSIGHT Cheminformatics Suite in Enalos Cloud Platform.

## 2. Methods

### 2.1. Data Collection and Curation

Acute oral toxicity data in rats were collected from three previously published studies by Chen et al. [46], da Silva and de Melo [47], and Lu et al. [48]. The original datasets were cross-referenced, and unique PFAS observations were compiled into a comprehensive dataset, including useful metadata (CAS numbers, SMILES representations, IUPAC names, and molecular weight (MW)), and the acute oral toxicity experimental values measured as the oral dose inducing death to 50% of the tested population. In all previous studies, the endpoint, LD_50_ (mg/kg), was initially transformed to LD_50_ (mol/kg) and, subsequently, to a more ML-friendly format through inverse logarithmic transformation (pLD_50_); however, in this analysis, the original measure units (mg/kg) were retained. In cases where the LD_50_ (mg/kg) values were not included in the source datasets, an additional search in the PubChem [52] database was conducted to extract the required information using the compounds’ CAS numbers.

Aiming to develop an interpretable ML model with a focus on the structural analysis of PFAS toxicity patterns, all PFAS were classified as high- or low-toxicity compounds based on their LD_50_ (mg/kg) values and the proposed toxicity thresholds by the EPA [53]. More precisely, only the Category III and Category IV compounds were included in the low-toxicity class, characterised by LD_50_ values higher than 500 mg/kg. Conversely, the Category I and Category II PFAS were assigned to the high-toxicity class. Moreover, dataset enrichment was achieved through the calculation of 777 one-dimensional (1D) and two-dimensional (2D) interpretable Mold2 molecular descriptors [54]. Morgan fingerprints were also evaluated as potential descriptors; however, modelling trials showed inferior performance, and therefore only Mold2 descriptors were used for further training (Table S1). The PFAS canonical SMILES representations were used as input in the in-house EnalosMold2 node [55] in the KNIME Analytics Platform [56], and the constitutional, count, graph invariants, vectorial, ring, and other descriptors were automatically generated. In this process, it was ensured that no PFAS mixtures or salts were included in descriptor calculations and modelling.

### 2.2. Data Preprocessing

**Figure 1:**
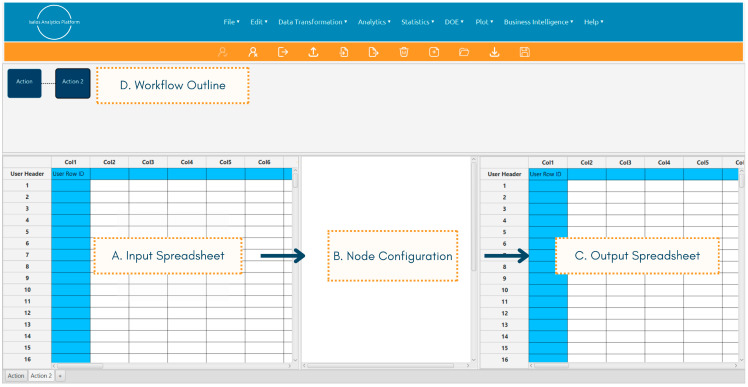
Isalos Analytics Platform graphical user interface, including (A) the input spreadsheet, (B) the node configuration section, (C) the output spreadsheet, and (D) the workflow outline in chart-like format.

Data preprocessing was exclusively conducted in the Isalos Analytics Platform, leveraging its enhanced capabilities, with the exception of a duplicate row check carried out in KNIME to mitigate the risk of model overfitting and limit bias in predictions. Isalos aims to democratise access to state-of-the-art methods in data analytics, ML, and design of experiments through an intuitive, spreadsheet-based environment that allows researchers of diverse backgrounds to conduct complex and demanding analyses regardless of previous coding experience. Each tab corresponds to a distinct node dedicated to a specific task, with interconnected tasks appearing in a chart-like format within the workflow outline section. Within each node, an input spreadsheet enables data import from previous steps, the configuration section supports analysis setup, and the resulting outputs are presented in an exportable output spreadsheet (Figure 1).

For this analysis, the complete dataset was imported into Isalos, and as an initial preprocessing step, the metadata columns were removed, retaining only the molecular descriptor features and the class variable. In the subsequent tab, the data were split into training and testing subsets using stratified random partitioning (70/30 ratio, seed = 590,729,676,119,100). The testing set, comprising 30% of the total data, was reserved exclusively for validation; therefore, all preprocessing steps from this point onward were applied only to the training set to avoid modelling bias. To reduce the high dimensionality of the feature space, columns containing more than 20% repeated values were filtered out. Further refinement was still required, however, to ensure model robustness and generalisability.

Feature selection was performed using the Boruta method, a wrapper approach particularly suitable for datasets in which the number of attributes exceeds the number of observations. This wrapper method builds upon the RF algorithm by iteratively randomising the attributes and repeatedly fitting RF models. Through a statistical test, a feature is deemed important if, across all iterations, the number of times its importance exceeds the maximum importance of the randomised features is significantly greater than the number expected by chance [57]. Using the embedded Boruta function, all features were normalised using the z-score method [58], and the default RF hyperparameter values were used for model development (feature fraction = 0.9, min impurity decrease = 0, and number of ensembles = 10). The procedure was executed for a maximum of 100 iterations.

### 2.3. AutoML-Based Optimisation and Modelling

The continuously evolving Isalos Analytics Platform has been enhanced to support automated ML model optimisation capabilities via the AutoML analytics option. The four ML algorithms commonly employed for classification applications, the kNN, XGBoost, RF, and NN models, were fine-tuned and evaluated within the AutoML workflow. Initially, the hyperparameter search ranges for each method were defined as shown in Table 1, and the grid search approach was employed to systematically explore the hyperparameter space. The number of nearest neighbours (k) was the only hyperparameter requiring tuning for the kNN method, while the XGBoost, RF, and NN models required adjustment of multiple parameters. In these cases, several parameters—such as the max tree depth, min impurity decrease, and the number of hidden neurons, respectively—were kept constant to decrease computational costs.

**Figure 2:**
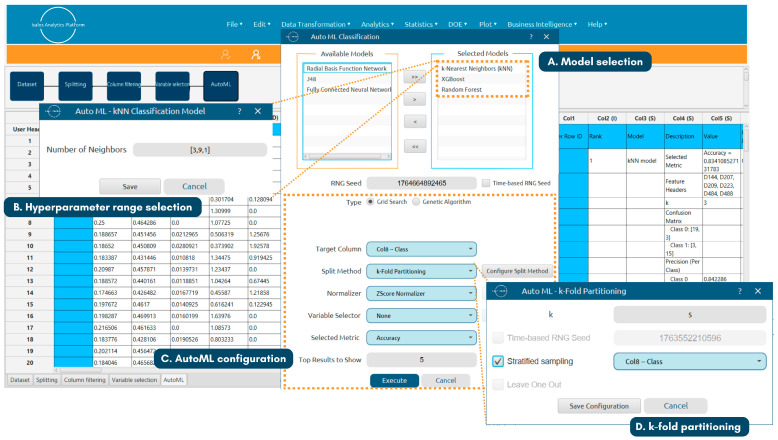
Isalos AutoML configuration for model fine-tuning. Steps shown include (A) model selection, (B) hyperparameter search range definition, (C) AutoML setup, and (D) k-fold partitioning selection.

Within the AutoML workflow, the training set used for model optimisation was normalised using the z-score method to maintain consistency with previous preprocessing steps. Candidate hyperparameter combinations for each model were evaluated via a stratified five-fold cross-validation scheme, in which the training set was partitioned into five folds while preserving the original high/low class ratio. Each fold was used once as a validation set per iteration, and the hyperparameter values yielding the highest average accuracy across the five folds were selected as optimal. The Isalos AutoML configuration is presented in Figure 2. The fine-tuned model resulting in the highest average accuracy was subsequently trained on the complete, normalised training set.

### 2.4. Model Validation

The optimal model was evaluated on the held-out testing set, which was normalised using the z-score parameters (mean and standard deviation) derived from the training set, through the Existing Model Utilisation offered by Isalos. Excluding the testing set from all preprocessing steps ensured an unbiased validation, maintaining statistical integrity and reflecting real-world performance. In addition to the accuracy (ACC, Equation (1)) statistic, which guided model and hyperparameter values selection, a series of commonly used statistical metrics were utilised for a more thorough evaluation. Therefore, the sensitivity (SEN, Equation (2)), specificity (SPE, Equation (3)), precision (PRE, Equation (4)), Matthews correlation coefficient (MCC, Equation (5)), and F1-score (Equation (6)) metrics were calculated.

(1) $$ACC=\frac{TP+TN}{TP+TN+FP+FN}$$

(2) $$SEN=\frac{TP}{TP+FN}$$

(3) $$SPE=\frac{TN}{TN+FP}$$

(4) $$PRE=\frac{TP}{TP+FP}$$

(5) $$MCC=\frac{TP\times TN-FP\times FN}{\sqrt{\left(TP+FP)(TP+FN)(TN+FP)(TN+FN\right)}}$$

(6) $$F1=\frac{2TP}{2TP+FP+FN}$$

where TPs are true positives, TNs are true negatives, FPs are false positives, and FNs are false negatives.

To further evaluate model stability with respect to data exclusion, a 10-fold cross-validation test was performed within the Isalos AutoML function. The training set was randomly split into 10 subsets, retaining the original high/low class ratio, and the optimal model was fitted 10 times, excluding a single fold once during each iteration and using it as a validation set. Consistency between the average evaluation metrics across all folds and the original performance values is indicative of good model stability and overall performance. Additional validation was achieved through y-randomisation, a technique in which the endpoint values are randomly permuted, and multiple models are fitted using the original input features but with shuffled target labels. In this study, 10 randomised models were developed (using the in-house *Y Randomization* node in KNIME v5.4.3) and used to make predictions on the testing set, after which the statistical metrics were recalculated. A significant decrease in the randomised models’ performance on the testing set, relative to the original model, is a strong indicator of the model’s robustness and its ability to capture meaningful relationships.

### 2.5. Applicability Domain

Keeping in line with OECD’s assessment of QSAR models framework, a clear definition of the model’s applicability domain (AD) is a requirement according to Principle 3: A defined applicability domain [59,60]. The AD is the data subspace inside which a prediction is based on interpolation, and is thus characterised as reliable [61]. Since multiple AD calculation methods exist, a Euclidean distance-based method offered by Isalos was used in this study. The Euclidean distances between all normalised training observations and their average value were calculated. For instances with distances lower than the average, a new average $\langle d\rangle$ and standard deviation (σ) value was computed and subsequently employed to define the AD threshold (thr) (Equation (7)).

(7) $$thr=\langle d\rangle +Z\sigma$$

The threshold represents the value over which an observation is characterised as unreliable, while Z is an empirical cutoff constant, set here to its default value of 0.5 [62].

### 2.6. Interpretability and PFAS Structure-Based Toxicity Analysis

Valuable insights into the relationship between the selected molecular descriptors and the PFAS toxicity class were extracted via a SHapley Additive exPlanation (SHAP) analysis, which is grounded on cooperative game theory [63]. SHAP analysis uses Shapley values to quantify each feature’s contribution towards the optimal model predictions. In this study, the model-agnostic Kernel Explainer was used to assign an importance value to each feature for every specific prediction made on the testing set and subsequently calculate the global importances [64]. The SHAP Python library (Python v.3.12.12, SHAP library v.0.50.0 in Google Colab environment, https://colab.research.google.com/, accessed on 23 January 2026) was used to compute Shapley values and visualise the results through a beeswarm plot, which provides a comprehensive overview of feature contributions. Features are displayed on the vertical axis in descending order of importance, consistent with the range of their Shapley values along the horizontal axis. The horizontal position of each point indicates the direction and magnitude of the feature’s effect: points to the left reflect negative contributions associated with lower endpoint values, while points to the right reflect positive contributions. It should be noted that the toxicity class variable was encoded as 0 (low toxicity) and 1 (high toxicity); therefore, points toward the right side of the plot indicate contributions to high predictions, whereas points on the left contribute to low-toxicity predictions [65].

**Figure 3:**
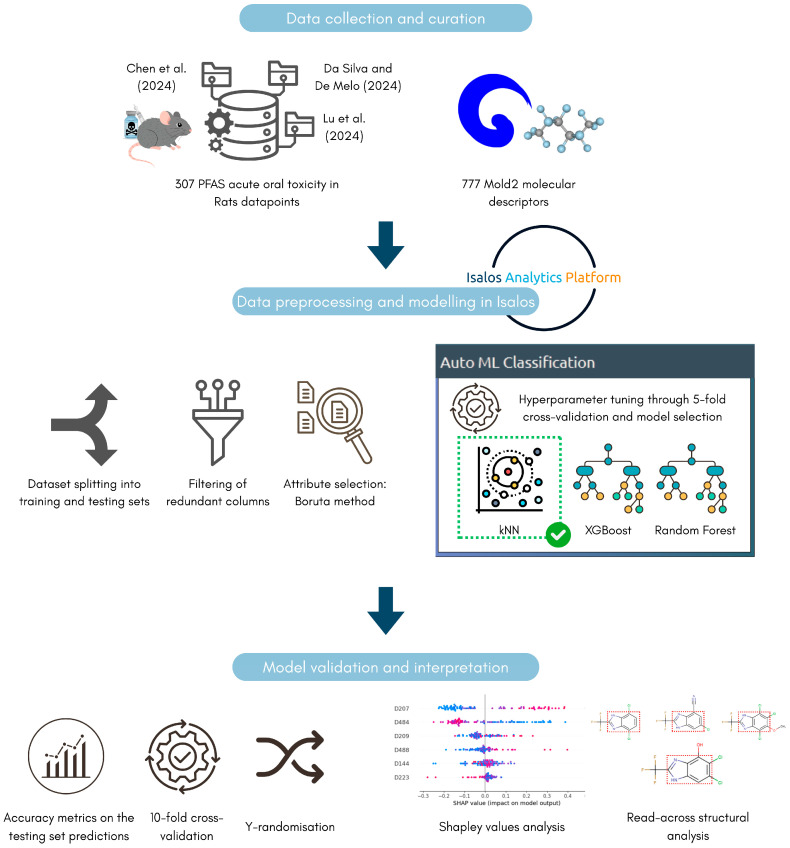
Analysis workflow schematically illustrating the key preprocessing, modelling, validation, and interpretation steps. Data was sourced from previous publications by Chen et al. [46], da Silva and de Melo [47], and Lu et al. [48], as shown in the figure.

To interpret the models’ classification decisions through the prism of chemical structure similarity, only the kNN model out of the tested algorithms enables instance-based, read-across analysis. Accordingly, the nearest neighbours of each compound were identified, and their structures were visualised to allow manual comparison with the reference PFAS. Key representatives of the different PFAS categories in the testing set were selected for this analysis. This approach allows for the identification of structural motifs and patterns shared between each reference PFAS and its nearest neighbours, providing insight into potential structure-toxicity relationships. The analysis leverages the molecular descriptor space used to train the kNN model, ensuring that structural similarity is assessed in a chemically meaningful context. The Euclidean distances between each PFAS compound and its nearest neighbours were also used as a qualitative measure for similarity assessment. The complete analysis workflow is presented in Figure 3.

## 3. Results

Aiming to elucidate the toxic effects of PFASs in rats, a robust analytical workflow was developed leveraging the advanced ML capabilities of Isalos Analytics Platform. A fundamental goal of this study was to derive meaningful relationships between the chemical structure of PFAS and their toxicity, bridging the knowledge gap between mechanistic understanding of chemical structures and predictive modelling.

### 3.1. Data Preprocessing and Attribute Selection

The compiled rat oral toxicity dataset comprised 307 observations classified into high- or low-toxicity classes based on their LD_50_ (mg/kg) values. The two classes were sufficiently balanced, with 195 high-toxicity and 112 low-toxicity compounds, and stratified random splitting into training and testing subsets (ratio 70/30) preserved the original class distribution (Table 2).

The training set—initially encompassing 215 PFAS compounds and 777 Mold2 molecular descriptors—was subsequently subjected to rigorous preprocessing. As a first step, 403 descriptors containing more than 20% repeated values were excluded. Variable selection through the Boruta method identified six variables whose importance in model development was significantly higher than their randomised counterparts (Table 3). Inter-correlations among the selected molecular descriptors were evaluated via the Pearson correlation coefficient, confirming that they do not elevate the risk of overfitting (Figure S1). The duplicate row filter revealed 4 redundant PFAS observations, which were removed, resulting in the final training set comprising 211 PFAS compounds and six selected attributes.

The selected molecular descriptors belong to the general descriptor categories of connectivity indices (D209, D207, D223), autocorrelation descriptors (D488, D484), and atomic volume descriptors (D144). Connectivity indices are derived from the molecular graph, offering a measure of the connectedness of the molecular structure. In particular, the average vertex connectivity descriptors used for modelling refer to the strength of the connection between atoms at 3-bonds (D209) and 1-bond (D207) distance, respectively, providing an indication of graph complexity at small-to-medium range and nearest-neighbour range distances. Additionally, through the valence connectivity index (D223), information about bonding and atom connection at larger distances of five bonds apart is captured. Moran autocorrelation descriptors are general spatial descriptors calculated from the 2D structure. In this case, D488 is a measure of atom size (volume) similarity at lengths of two bonds apart, while D488 captures longer-range (six length distances) mass distribution motives. Positive numbers of these descriptors indicate the presence of similar molecular geometry patterns at the specified molecular distances. Finally, D144 is a count of the van der Waals volumes of carbon atoms within the molecule, offering a useful measure of size differences between PFAS structures [66,67].

The selection of a carbon-centric descriptor (D144) may introduce a bias toward PFAS chain length, potentially reducing generalizability for atypical structures in which fluorination patterns significantly influence toxicity. This limitation could be addressed by incorporating fluorine-specific molecular descriptors to enhance both prediction accuracy and mechanistic interpretability. However, in this study, all descriptors capturing fluorine atom contributions were removed during initial preprocessing as redundant (containing more than 20% repeated values). Consequently, within this dataset, the included PFAS structures do not vary substantially based on fluorine-driven features, and forcing the inclusion of these descriptors could compromise model robustness without providing additional predictive value. Moreover, the inclusion of two autocorrelation descriptors derived from the 2D molecular graph—capturing fluorine atom information—partially mitigates this bias. In future work, efforts should focus on enhancing the dataset with more diverse PFAS structures and enriching the descriptors to capture broader molecular features and provide greater insight into toxicity mechanisms.

### 3.2. AutoML-Based Optimisation and Model Selection

A kNN, XGBoost, RF, and NN model were optimised using grid search combined with five-fold cross-validation on the training set, with the average accuracy across folds guiding the selection of the best-performing models (Table 4). Consistent with the computationally efficient design of the Isalos platform, only the most critical hyperparameters were available to be optimised for the kNN and RF models, yet the high predictive accuracy of the resulting models indicates that this limited tuning was sufficient.

Although all four algorithms achieved closely comparable performance, the kNN model attained the highest average cross-validated accuracy. In addition, a central aim of this study was to enable structural interpretation of PFAS toxicity and to elucidate potential mechanistic factors influencing acute oral toxicity. A neighbour-based, read-across model is particularly suitable for this purpose, as it facilitates the identification of structurally related compounds under ML rules while integrating computational and expert-driven interpretation. Integrating the kNN method into the read-across framework automatically generates a data-driven similarity group for each compound, consisting of its *k* nearest neighbours in the descriptor space. The toxicity prediction is therefore based on these algorithmically defined neighbours rather than on manually constructed similarity groups derived from human-selected criteria [68,69]. For these reasons, the kNN model was selected as both the top-performing algorithm and the most appropriate framework for structure-informed toxicity prediction in rats.

### 3.3. Model Validation and Applicability Domain

**Figure 4:**
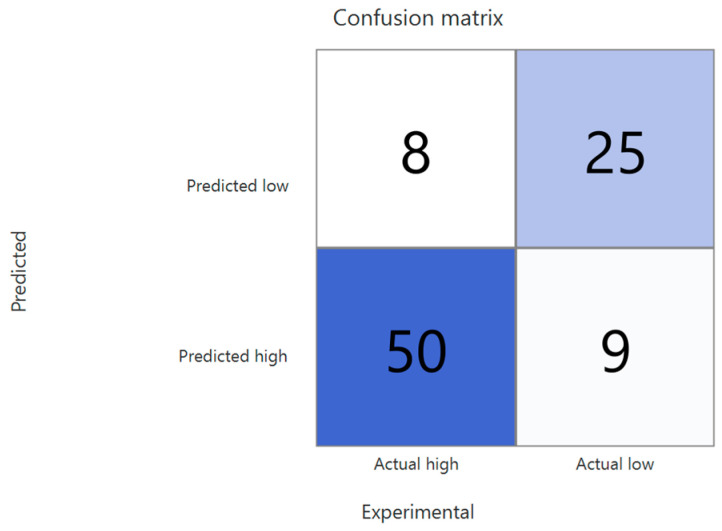
Confusion matrix for the testing set predictions, with true positive and true negative classifications highlighted in the colour blue.

The optimised kNN model was validated through common statistical metrics derived from the confusion matrix—visualised through the Business Intelligence functionalities offered by Isalos (Figure 4). The high accuracy achieved on the testing set demonstrates the strong predictive capability of the model. Furthermore, the high sensitivity, specificity, and MCC values indicate a solid ability to distinguish between the high- and low-toxicity classes, performing well across both classes. The comparatively lower sensitivity value suggests a subtly reduced ability to correctly classify low-toxicity instances, which is expected given the slight underrepresentation of this class in the training set (the classification metrics are presented in Table 5). Nevertheless, the overall performance of the model remains highly robust.

A 10-fold cross-validation on the training set was performed as an internal validation step to assess the optimised model’s robustness and stability against data exclusion. In this case, the reported statistical metrics are calculated as the average value across all folds (Table 6). Cross-validation yielded slightly higher metric values compared to the held-out testing set evaluation. Nonetheless, the similarity between the metrics in both cases indicates model stability across different training set partitioning as well as a strong generalisability potential.

The y-randomisation analysis also confirmed a strong relationship between the selected molecular descriptors and the toxicity endpoint, as the permuted models performed markedly worse than the original kNN model. The substantial decrease in all performance metrics highlights the randomness of predictions generated from shuffled endpoints. In particular, among the metrics capturing the overall performance of the prediction model, an accuracy value approaching 0.5 and an MCC value near 0 indicate a complete lack of correlation between the input data and the permuted toxicity labels (Table 7).

Model assessment was completed by defining the model’s AD in accordance with OECD guidelines. The threshold for determining when a new observation falls outside the AD—and therefore yields an unreliable prediction—was calculated as 2.149. Among the 92 toxicity predictions in the testing set, only one was classified as unreliable (Table 8).

### 3.4. SHAP Analysis Insights

**Figure 5:**
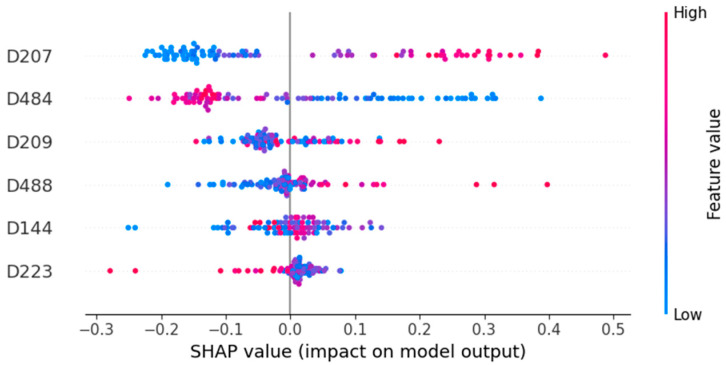
SHAP analysis results in the form of a beeswarm plot. Red-coloured points represent high values for the respective variable shown in the vertical axis, while blue-coloured points represent low values. The position of the data points at the left or right of the zero-SHAP value line indicates a relationship with low- and high- toxicity class predictions, respectively.

The SHAP analysis revealed the important role and direction of contribution of the selected molecular connectivity and autocorrelation descriptors (Figure 5). The connectivity indices D207 and D209, calculated at small-range distances of one and three bonds apart, respectively, contributed significantly to toxicity predictions, with their highest values (red-coloured points in the SHAP plot) pushing the predictions towards the high-toxicity class. This effect was most pronounced for D207, which reflects nearest-neighbour connectivity, whereas D209—capturing connectivity at a three-bond range—showed the same trend but with a greater number of exceptions. Connectivity indices have been extensively studied as indicators of chemical toxicity [70] and have already shown a positive correlation between increased structural complexity or density and QSAR-based predictions of high acute oral toxicity [71].

On the other hand, the longer-range (five-bonds-apart distance) valence connectivity descriptor D223 had the lowest importance to toxicity predictions, nonetheless showing a strong correlation between its highest values and the low-toxicity class. Mechanistically, less localised electron density and more equally distributed longer-range valence connectivity could signify more stable, less reactive compounds, thus leading to low-toxicity predictions. As valence connectivity indices have shown strong correlations with toxicity endpoints, they have been extensively incorporated in QSAR models over the past few decades. Earlier studies reported a positive relationship between short-range connectivity descriptors (zero- or first-order) and chemical toxicity [72,73]. However, more recent studies have highlighted an inverse relationship between higher-order valence connectivity indices and human acute toxicity [74].

Moran topological structure autocorrelation descriptors followed closely the vertex connectivity indices in the SHAP-based importance ranking, confirming their previously observed significance in QSAR toxicity models [75,76]. The autocorrelation descriptor weighted by atomic masses and calculated at medium-to-long (six-bond) distances (D484) showed a strong negative relationship with the high-toxicity class. Similar trends have been reported in a previous study, where long-range atomic-mass-weighted autocorrelation descriptors were negatively associated with the acute inhalation toxicity of herbicides to sheepshead minnow, whereas the opposite relationship was observed for the short-range descriptor in models of fungicide toxicity [77]. In this work, high values of the Moran autocorrelation descriptor D488, which captures the van der Waals volume distribution at two-bond distances, lead to high-toxicity predictions. It is indicated, therefore, that a more balanced and extended mass distribution across the molecular structure at long atomic distances correlates with reduced toxicity effects. In contrast, a bulkier geometry at small atomic distances seems to enhance the PFAS compound toxicity.

Finally, D144 was the only descriptor for which SHAP analysis results were not interpretable. While there have been indications of a correlation between mean atomic van der Waals volume (carbon-scale) and lower acute oral toxicity predictions [78], in this case, its high and low values appeared on both sides of the SHAP plot, preventing the identification of a clear relationship with the toxicity class. The limited interpretability likely reflects the carbon-centric nature of the D144 descriptor, which omits information on toxicity-relevant structural features, including fluorine atoms and terminal functional groups (e.g., carboxylate and sulfonate moieties). To address this limitation and enhance mechanistic interpretability, future studies should aim to incorporate fluorine-specific and functional-group descriptors, while preserving model predictive performance and robustness.

## 4. Discussion

Following thorough validation, model predictions for representative testing set compounds were evaluated against both experimental data and the results of existing prediction tools to confirm modelling accuracy prior to structural analysis. Upon completion of this step, the model enabled read-across analysis based on structural similarity, consistent with the principles of explainable machine learning. This approach provided detailed insights into the molecular and geometrical features of PFASs that contribute to elevated acute oral toxicity.

### 4.1. Benchmarking of Predictions Prior to Structural Analysis

The developed model is specialised for PFAS acute oral toxicity predictions; however, more general in silico tools are also available (e.g., CATMoS [37], TEST [38], ProTox [45]), as noted previously. To benchmark performance, four PFASs from the testing set were selected for comparative predictions using our model and the webTEST service [38]. Three high-toxicity and one low-toxicity PFAS were submitted to webTEST via their CAS numbers to obtain LD_50_ predictions, which were then compared with experimental values. Both models correctly classified the PFAS toxicity classes, although webTEST showed notable deviations in the numerical LD_50_ values (Table 9). This analysis demonstrates that our classification-based predictions are consistent with results from broader tools while offering greater reliability, minimising potential errors that can arise in regression-based LD_50_ predictions.

### 4.2. Read-Across Structural Analysis

**Figure 6:**
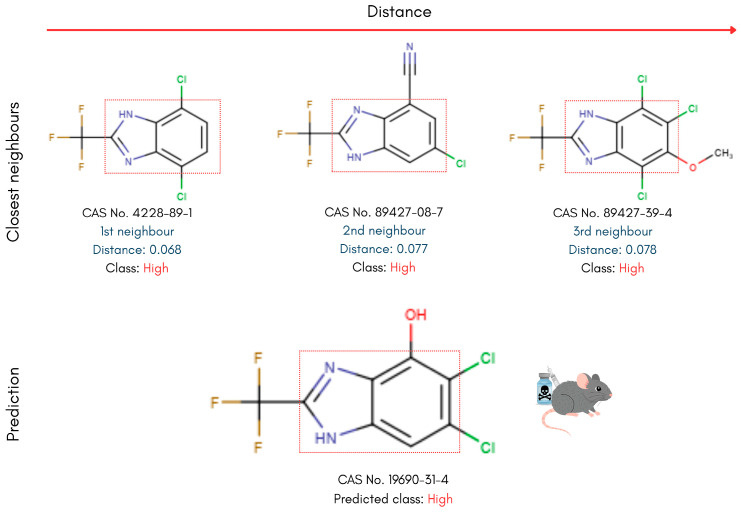
Read-across structural insights for high acute oral toxicity PFAS consisting of an aromatic bicyclic backbone.

**Figure 7:**
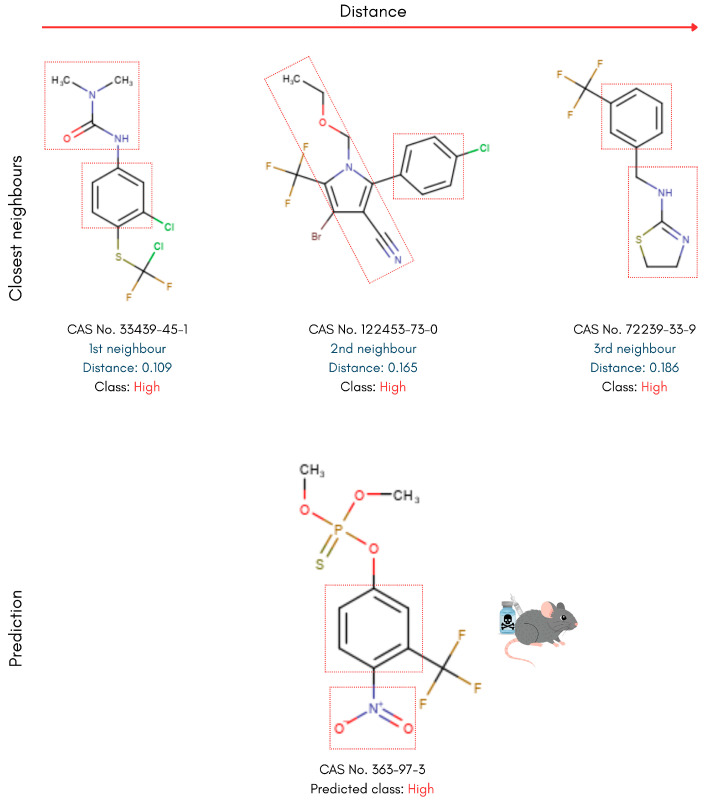
Read-across structural insights for high acute oral toxicity PFASs consisting of a benzene-ring scaffold and a nitrogen-containing heteroatom sidechain.

**Figure 8:**
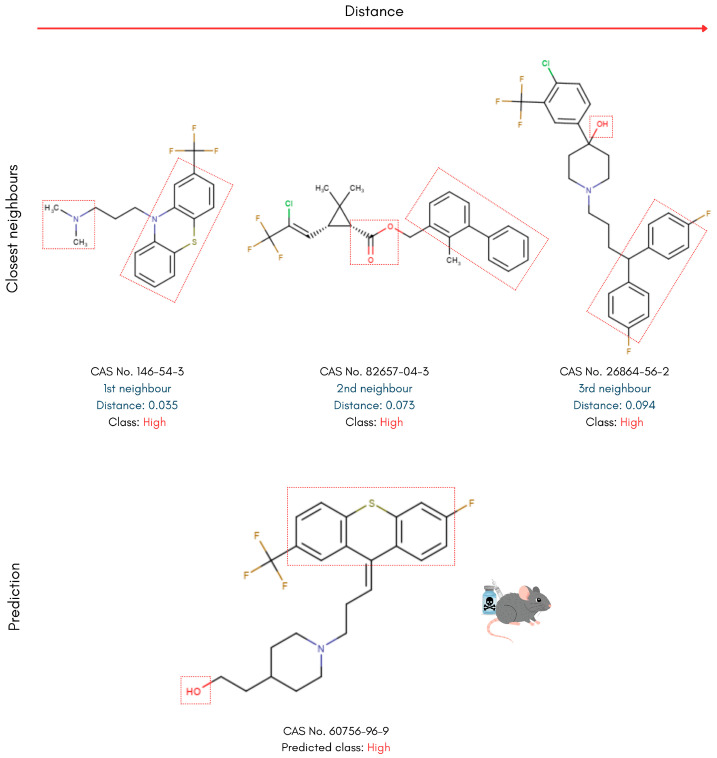
Read-across structural insights for high acute oral toxicity PFAS, consisting of a ring-rich domain and a polar end-group separated by a long linker.

**Figure 9:**
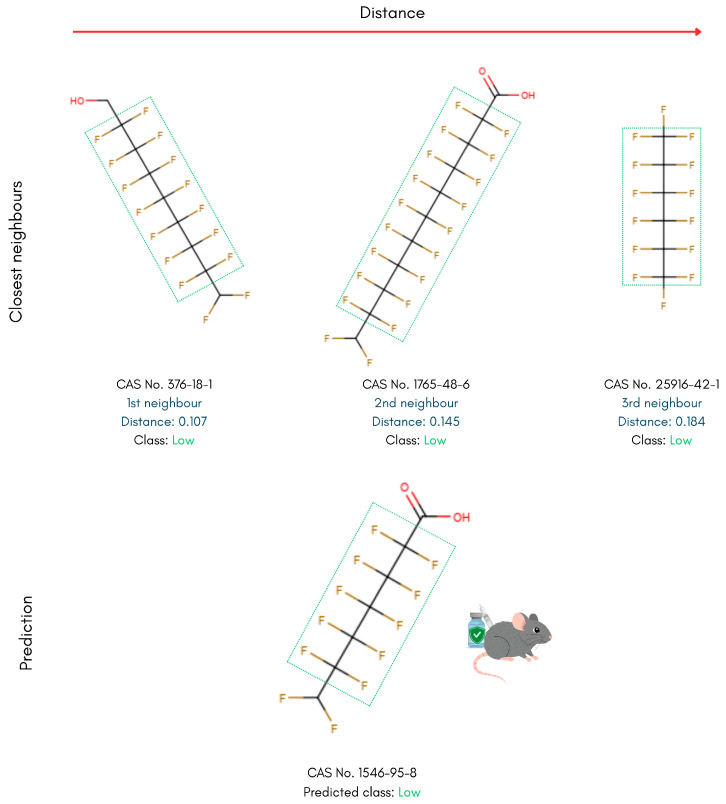
Read-across structural insights for low acute oral toxicity, linear PFASs.

Building on the kNN-based read-across approach, PFAS in the testing set were examined alongside their nearest neighbours in the training set to interpret the model’s predictions. Diverse compounds used for toxicity prediction benchmarking were analysed to reveal molecular geometries and functional groups that distinguish high- from low-toxicity PFASs. In the following examples, the three nearest neighbours are displayed at the top alongside their calculated distances, with the reference test set PFAS displayed below (Figure 6, Figure 7, Figure 8 and Figure 9).

Compounds containing aromatic bicyclic structures were most consistently classified as high-toxicity PFASs. For example, the prediction of high acute oral toxicity for 5,6-dichloro-2-(trifluoromethyl)-1H-benzimidazol-4-ol (CAS 19690-31-4) was unanimous, supported by the classifications of its three nearest neighbours in the training set, each exhibiting pronounced structural similarity (Figure 6). Both the reference compound and its training set neighbours are characterised by a fused-ring backbone consisting of a benzene and an imidazole ring. This benzimidazole moiety in their core scaffold modulates electronic distribution through its heteroaromatic nitrogen atoms and provides a rigid, planar framework. The delocalised electrons in these aromatic systems confer enhanced structural stability, increase hydrophobicity, and can influence biological activity, which may thus account for the high-toxicity prediction produced by the model [79,80,81,82]. Furthermore, halogen (Cl) substitution on the benzene ring observed across all studied PFAS in this category enhances the electron-withdrawing character of the chemicals and, therefore, their hydrophobicity and bioaccumulation potential.

To further study the structural motifs associated with high-toxicity predictions, a reference PFAS molecule exhibiting greater structural diversity relative to its nearest neighbours was selected. Dimethoxy-[4-nitro-3-(trifluoromethyl)phenoxy]-sulfanylidene-λ^5^-phosphane (CAS 363-97-3) was accurately classified as a high-toxicity chemical based on the class of its three nearest neighbours, which, however, exhibited larger neighbour distances compared with the previous example (Figure 7). All structures incorporate a phenyl ring in their core scaffold, which greatly contributes towards planarity and π-stacking/hydrophobic character, as well as structural similarity and, possibly, compatibility with similar binding sites or toxic pathways. A more subtle but recurring motif across the reference structure and its nearest neighbours is the presence of nitrogen-containing functional groups positioned close to the core aromatic ring. These structures can participate in hydrogen-bonding interactions, increasing metabolic reactivity and contributing to the model’s assignment of higher acute oral toxicity. However, the more evident structural similarity—reflected by the smallest neighbour distance of 0.109—between the reference PFAS molecule and its closest neighbour is reinforced by the presence of comparable nitrogen-containing side chains, with simpler urea-like or amine-like substituents rather than fully cyclic heterocycles.

To further illustrate how the model captures the structural diversity of highly toxic PFAS, a more complex molecule—cis-piflutixol (CAS 60756-96-9)—was selected for detailed structural analysis. In the chemical descriptor space, this compound lies close to three PFASs that are diverse in overall shape, yet share clear toxicity-related structural motifs (Figure 8). All compounds are characterised by a polyaromatic backbone, containing two or more cyclic structures. Aromatic bulk, as mentioned before, can be correlated with high lipophilicity, leading to reduced excretion rates from biological systems and intensified toxic effects. Additionally, the hydrophobic, ring-rich domains of the reference PFAS molecule and its first and third closest neighbours are separated from their polar functional groups (such as tertiary amines or alcohols) by relatively long linkers with aliphatic characteristics. This arrangement produces an amphiphilic structural pattern that can enhance bioavailability and membrane permeability, which may in turn be associated with increased toxicity.

To conclude the read-across structural analysis, a representative example from the low-toxicity class was examined to highlight inter-class differences. The short-chain, aliphatic PFAS 2,2,3,3,4,4,5,5,6,6,7,7-dodecafluoroheptanoic acid (CAS 1546-95-8), which was correctly assigned to the low acute oral toxicity class, and its three nearest neighbours in descriptor space exhibited similarly linear geometries (Figure 9). Across all four structures, the absence of aromatic rings and additional heteroatoms is notable and aligns with their lower-toxicity assignments. Moreover, the reference compound and its two closest neighbours contain small polar terminal groups (carboxylic acid or primary alcohol), further distinguishing them from the structural motifs associated with the high-toxicity class.

Overall, although PFAS structure–toxicity relationships are yet to be fully explored, the present read-across analysis suggests that polyaromatic, heterocyclic PFASs may be associated with higher acute oral toxicity compared with simpler, less bulky linear structures. These differences may relate to altered lipophilicity or, possibly, distinct molecular interaction pathways. Indeed, while systematic experimental data are limited, there are available indications pointing to a potential contribution of polyaromatic or heterocyclic moieties to elevated toxicity [83].

### 4.3. Data and Model Dissemination

Following the Findability, Accessibility, Interoperability, and Reusability (FAIR) principles [84], the complete modelling dataset—along with a brief description of its key components—is made accessible via the freely available ChemPharos database (https://db.chempharos.eu/datasets/Datasets.zul?datasetID=ds17, accessed on 23 January 2026), which hosts multiple ready-to-model datasets for the prediction of small molecules’ propertie. Promoting modelling transparency, the standardised QSAR Model Reporting Format (QMRF) was followed to report the modelling and validation methods used in this study (Table S2).

**Figure 10:**
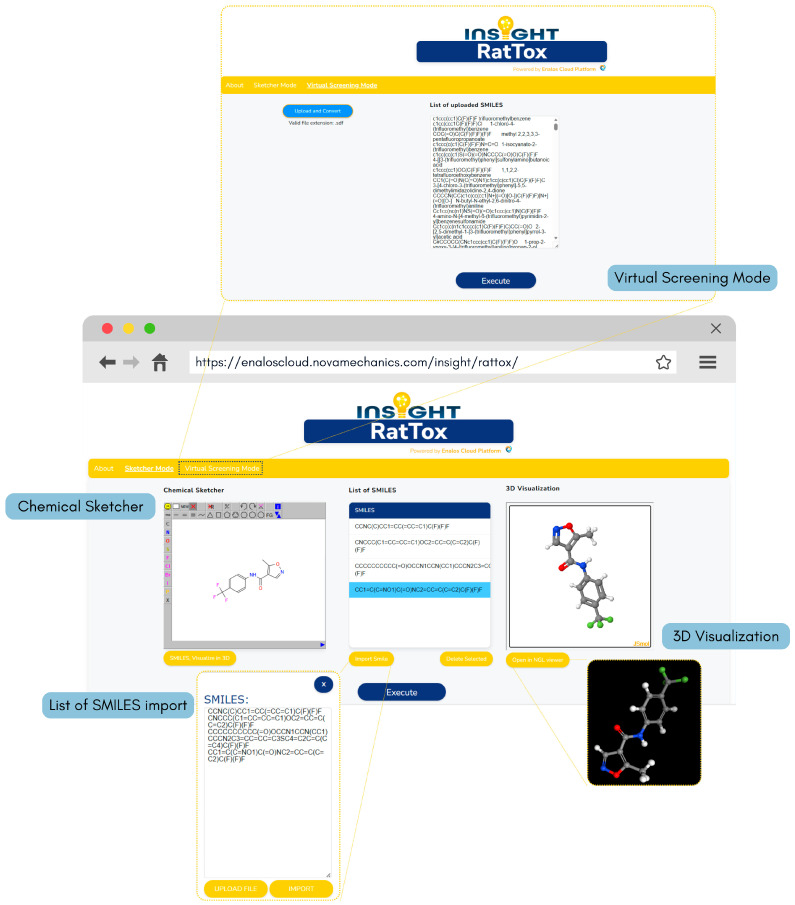
INSIGHT RatTox web service graphical user interface, highlighting the Chemical Sketcher, List of SMILES, 3D Visualisation, and Virtual Screening Mode components.

**Figure 11:**
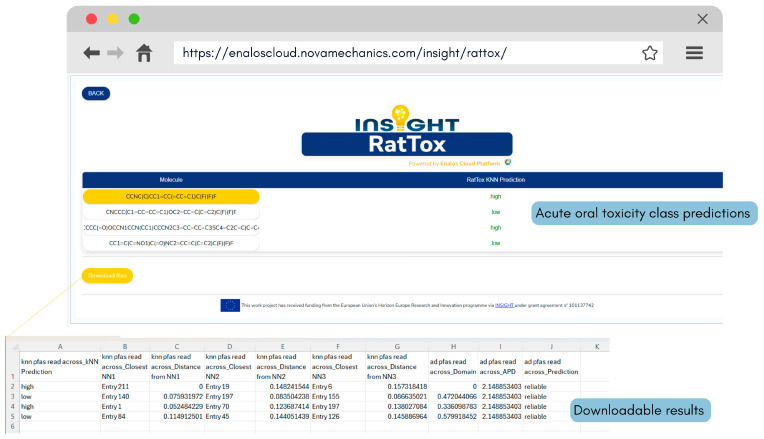
INSIGHT RatTox prediction results showing the predicted acute oral toxicity class (high or low), colour-coded according to the model applicability domain (green for reliable and red for unreliable predictions). The downloadable results also include the nearest neighbours identified by the algorithm and their distances from the predicted compound in the descriptor space.

Additionally, the optimised kNN model is distributed as the INSIGHT RatTox web application (https://enaloscloud.novamechanics.com/insight/rattox/, accessed on 23 January 2026) and is also included in the INSIGHT Cheminformatics Suite (https://www.enaloscloud.novamechanics.com/insight/cheminf/, accessed on 23 January 2026) offered by the Enalos Cloud Platform. Within the RatTox open access web service, new chemical compounds can be drawn in the “Chemical Sketcher” section or imported in SMILES format with the option to visualise imported molecules in the “3D Visualizer” window. Batch screening is also supported through the “Virtual Screening Mode,” which allows users to upload an SDF file containing multiple PFAS SMILES for prediction (Figure 10). Upon clicking “Execute”, predictions are generated automatically along with a colour-coded indication of their reliability based on the models’ pre-defined ADs (the green colour denotes reliable predictions while the red colour denotes unreliable predictions). The downloadable results for kNN-based predictions also include the nearest neighbours identified by the algorithm and their distances from the reference compound in the descriptor space, allowing users to fully supervise the model’s decision processes (Figure 11).

## 5. Conclusions

Human-made chemicals released through diverse industrial or everyday activities have been shown to exert detrimental effects on both ecosystems and human health. Among the most prominent chemical pollutants are PFASs, exposure to which has been linked to hepatotoxic, developmental, and neurotoxic outcomes, among others. Global initiatives for the long-term phase-out of PFASs are underway; however, current scientific efforts are directed toward the more attainable goal of understanding PFAS toxicity mechanisms and leveraging this knowledge to identify low-toxicity emerging PFASs for critical applications.

Contributing to the development of in silico approaches for high-throughput and resource-efficient screening of PFASs, we developed an accurate read-across model for the prediction of PFAS acute oral toxicity class (high or low) in rats, using selected molecular descriptors derived from PFAS chemical structure. The advanced ML capabilities of Isalos Analytics Platform were used to optimise four ML models: a kNN, RF, XGBoost, and NN model. The kNN model (k = 3), achieving the highest accuracy score in five-fold cross-validation, was selected as the final predictive model. When applied to the held-out testing set, it yielded an accuracy of 81.5%. Additional evaluation, including 10-fold cross-validation and y-randomisation, demonstrated excellent model stability against data exclusion and strong robustness. The model was also used to extract mechanistic insights into the influence of the selected descriptors on predicted toxicity class through SHAP analysis. Importantly, the read-across methodology enabled investigation of distance-based similarity groups—comprising each reference PFAS and its three closest neighbours in the training set—providing meaningful structural information potentially associated with higher PFAS acute oral toxicity.

The SHAP analysis indicates that bulky, densely packed structures at short atomic ranges are potential markers of high toxicity, whereas a more balanced mass and electron density distribution at larger atomic ranges shows the opposite association. This study also highlighted the need to include fluorine-specific and terminal functional group descriptors in future work to enhance mechanistic interpretability. Furthermore, read-across structural analysis of the representative testing set PFASs identified polyaromatic, heterocyclic moieties in the PFAS structure as contributors to higher toxicity, in contrast to simpler linear PFAS molecules lacking heteroatoms. Overall, the developed read-across model—freely available through the Enalos Cloud Platform—demonstrates strong predictive performance for the PFAS acute oral toxicity class while providing transparency into the model’s decision process. The combination of high predictive accuracy and the ability to examine molecular similarity patterns within k-nearest-neighbour groups offers a valuable tool for screening and identifying existing or emerging PFAS with lower toxicity profiles.

## Figures and Tables

**Table 1:** Hyperparameter search ranges for model optimisation inside the Isalos AutoML scheme.

ML Method	Hyperparameter	Search Range [Min, Max; Step]
kNN	Number of nearest neighbours, k	[3, 9; 1]
XGBoost	Number of trees	[5, 20; 5]
	Learning rate	[0.1, 0.3; 0.1]
	Gamma (constant)	[0, 0; 1]
	Max tree depth (constant)	[6, 6; 1]
	Minimum child weight (constant)	[1, 1; 1]
	Column sample by tree (constant)	[1, 1; 1]
	Subsample (constant)	[1, 1; 1]
	Lambda	[0.8, 1.0; 0.1]
	Alpha	[0.8, 1.0; 0.1]
Random Forest	Feature fracture	[0.5, 0.9; 0.2]
	Min impurity decrease (constant)	[0, 0; 1]
	Number of ensembles	[100, 200; 50]
Fully Connected Neural Network	Number of hidden layers (constant)	[2, 2; 1]
	Number of neurons/layer	[50, 100, 50]
	Activation function	RELU
	Batch size (constant)	[128, 128; 1]
	Number of epochs	[50, 150; 50]
	Learning rate	[0.001; 0.01; 0.009]
	Momentum	[0.8, 0.9; 0.1]

**Table 2:** Class distribution in the training and testing sets.

Subset	Low Toxicity	High Toxicity
Training	36%	64%
Testing	37%	63%

**Table 3:** List of molecular descriptors selected for modelling, along with a brief explanation.

Molecular Descriptor	Explanation
D209	Average vertex connectivity order-3 index.
D207	Average vertex connectivity order-1 index.
D223	Average valence vertex connectivity order-5 index.
D488	Moran topological structure autocorrelation length-2 weighted by atomic van der Waals volumes.
D484	Moran topological structure autocorrelation length-6 weighted by atomic masses.
D144	Mean atomic van der Waals carbon-scale.

**Table 4:** AutoML optimisation results of a kNN, XGBoost, RF, and NN model. The optimal hyperparameters are reported along with the maximised average accuracy statistic achieved during cross-validation.

ML Method	Hyperparameter	Optimal Value	5-Fold Accuracy
kNN	Number of nearest neighbours, k	3	0.834
XGBoost	Number of trees	20	0.829
	Learning rate	0.2	
	Gamma	0	
	Max tree depth	6	
	Minimum child weight	1	
	Column sample by tree	1	
	Subsample	1	
	Lambda	1	
	Alpha	0.8	
Random Forest	Features fracture	0.7	0.829
	Min impurity decrease	0	
	Number of ensembles	150	
Fully Connected Neural Network	Number of hidden layers	2	0.824
	Number of neurons/layer	100	
	Activation function	RELU	
	Batch size	128	
	Number of epochs	0.01	
	Learning rate	0.9	

**Table 5:** Statistical metrics capturing the model’s performance on the testing set.

Metric	Accuracy	Precision	Sensitivity	Specificity	F1-Score	MCC
Value	0.815	0.758	0.735	0.862	0.746	0.601

**Table 6:** Statistical metrics achieved during 10-fold cross-validation on the training set.

Metric	Accuracy	Precision	Sensitivity	Specificity	F1-Score	MCC
Value	0.829	0.773	0.824	0.811	0.794	0.623

**Table 7:** Y-randomisation results, presented as the average accuracy and MCC values across 10 iterations, accompanied by their standard deviation (σ).

Metric	Accuracy	MCC
Value ± σ	0.568 ± 0.039	0.041 ± 0.097

**Table 8:** AD threshold and percentage of reliable predictions on the testing set.

AD Threshold	Reliable Predictions
2.149	98.9%

**Table 9:** Comparison of selected testing set toxicity predictions (read-across kNN) with the webTEST tool and experimental data.

CAS Number	Read-Across kNN	webTEST [38]	Experimental
19690-31-4	High	High (11.262 mg/kg)	High (52 mg/kg)
363-97-3	High	High (199.588 mg/kg)	High (250 mg/kg)
60756-96-9	High	High (11.527 mg/kg)	High (1.5 mg/kg)
1546-95-8	Low	Low (807.052 mg/kg)	Low (518 mg/kg)

## Data Availability

The data presented in this study are available on the following addresses: **Enalos Cloud web application**: INSIGHT: RatTox. (https://enaloscloud.novamechanics.com/insight/rattox/, accessed on 23 January 2026). **APIs**: Swagger UI. (https://enaloscloud.novamechanics.com/insight/swagger-ui/index.html, accessed on 23 January 2026). **Dataset**: ChemPharos Dataset Query Page. (https://db.chempharos.eu/datasets/Datasets.zul?datasetID=ds17, accessed on 23 January 2026).

## Supplementary Materials

The following supporting information can be downloaded at: https://www.mdpi.com/article/10.3390/toxics14020152/s1. Figure S1: Pearson correlation heatmap of the selected input Mold2 descriptors; Table S1: AutoML optimisation results of a kNN, XGBoost, RF, and NN model trained on the Morgan fingerprints descriptor set; Table S2: Rat PFAS-LD50 kNN/read-across –(Q)SAR model reporting format (QMRF) v.2.1.

## Abbreviations

*ACC*	Accuracy
*AD*	Applicability domain
*AutoML*	Automated machine learning
*CAS*	Chemical Abstracts Service
*CATMoS*	Collaborative Acute Toxicity Modelling Suite
*EPA*	Environmental Protection Agency
*EU*	European Union
*FAIR*	Findability, Accessibility, Interoperability, and Reusability
*FN*	False negative
*FP*	False positive
*kNN*	k-nearest neighbours
*LC*_50_	Acute inhalation toxicity
*LD*_50_	Acute oral toxicity
*MCC*	Matthews correlation coefficient
*ML*	Machine learning
*MW*	Molecular weight
*NN*	Neural network
*OECD*	Organisation for Economic Co-operation and Development
*PFASs*	Per- and polyfluoroalkyl substances
*PFHxS*	Perfluorohexane sulfonate
*PFOA*	Perfluorooctanoic acid
*PFOS*	Perfluorooctane sulfonate
*PRE*	Precision
*QMRF*	QSAR Model Reporting Format
*QSAR*	Quantitative structure–activity relationship
*RF*	Random forest
*ROS*	Reactive oxygen species
*SDF*	Simple data format
*SEN*	Sensitivity
*SHAP*	SHapley Additive exPlanations
*SMILES*	Simplified molecular input line entry specification
*SPE*	Specificity
*TEST*	Toxicity Estimation Software Tool
*TN*	True negative
*TP*	True positive
*U.S.*	United States
*WHO*	World Health Organization
*XGBoost*	eXtreme Gradient Boosting

## References

[1] Ellis E.C., Magliocca N.R., Stevens C.J., Fuller D.Q. (2018). Evolving the Anthropocene: Linking Multi-Level Selection with Long-Term Social–Ecological Change. Sustain. Sci..

[2] Shetty S.S., Deepthi D., Harshitha S., Sonkusare S., Naik P.B., Kumari N.S., Madhyastha H. (2023). Environmental Pollutants and Their Effects on Human Health. Heliyon.

[3] Manisalidis I., Stavropoulou E., Stavropoulos A., Bezirtzoglou E. (2020). Environmental and Health Impacts of Air Pollution: A Review. Front. Public Health.

[4] World Health Organization (WHO) (2019). Health, Environment and Climate Change. Seventy-Second World Health Assembly.

[5] Prevedouros K., Cousins I.T., Buck R.C., Korzeniowski S.H. (2006). Sources, Fate and Transport of Perfluorocarboxylates. Environ. Sci. Technol..

[6] Schröter-Kermani C., Müller J., Jürling H., Conrad A., Schulte C. (2013). Retrospective Monitoring of Perfluorocarboxylates and Perfluorosulfonates in Human Plasma Archived by the German Environmental Specimen Bank. Int. J. Hyg. Environ. Health.

[7] Secundo L., Metrangolo P., Dichiarante V. (2025). Current Approaches in the Classification of PFAS: An Overview. Chem. Asian J..

[8] Hammel E., Webster T.F., Gurney R., Heiger-Bernays W. (2022). Implications of PFAS Definitions Using Fluorinated Pharmaceuticals. iScience.

[9] OECD (2021). Reconciling Terminology of the Universe of Per- and Polyfluoroalkyl Substances: Recommendations and Practical Guidance.

[10] Schymanski E.L., Zhang J., Thiessen P.A., Chirsir P., Kondic T., Bolton E.E. (2023). Per- and Polyfluoroalkyl Substances (PFAS) in PubChem: 7 Million and Growing. Environ. Sci. Technol..

[11] Mintis D.G., Papavasiliou C., Varsou D.-D., Tsoumanis A., Melagraki G., Seif J.P., Majó M., Del Real A.J., Serchi T., Hischier R. (2026). SimpleBox4Planet: Environmental Fate Modelling of PFAS and Their Alternatives via the Enalos Cloud Platform. RSC Sustain..

[12] Alam M.J., Habib A., Hasan M.M., Islam S., Halim E. (2025). Industrial Applications, Environmental Fate, Human Exposure, and Health Effects of PFAS. Pollutants.

[13] Habib Z., Song M., Ikram S., Zahra Z. (2024). Overview of Per- and Polyfluoroalkyl Substances (PFAS), Their Applications, Sources, and Potential Impacts on Human Health. Pollutants.

[14] Meegoda J.N., Kewalramani J.A., Li B., Marsh R.W. (2020). A Review of the Applications, Environmental Release, and Remediation Technologies of Per- and Polyfluoroalkyl Substances. Int. J. Environ. Res. Public Health.

[15] Louisse J., Rijkers D., Stoopen G., Janssen A., Staats M., Hoogenboom R., Kersten S., Peijnenburg A. (2020). Perfluorooctanoic Acid (PFOA), Perfluorooctane Sulfonic Acid (PFOS), and Perfluorononanoic Acid (PFNA) Increase Triglyceride Levels and Decrease Cholesterogenic Gene Expression in Human HepaRG Liver Cells. Arch. Toxicol..

[16] Ehrlich V., Bil W., Vandebriel R., Granum B., Luijten M., Lindeman B., Grandjean P., Kaiser A.-M., Hauzenberger I., Hartmann C. (2023). Consideration of Pathways for Immunotoxicity of Per- and Polyfluoroalkyl Substances (PFAS). Environ. Health.

[17] Zeng H., Zhu B., Wang Y., He Q. (2021). ROS-Triggered Autophagy Is Involved in PFOS-Induced Apoptosis of Human Embryo Liver L-02 Cells. BioMed Res. Int..

[18] Li X., Li T., Wang Z., Wei J., Liu J., Zhang Y., Zhao Z. (2021). Distribution of Perfluorooctane Sulfonate in Mice and Its Effect on Liver Lipidomic. Talanta.

[19] Ramhøj L., Hass U., Gilbert M.E., Wood C., Svingen T., Usai D., Vinggaard A.M., Mandrup K., Axelstad M. (2020). Evaluating Thyroid Hormone Disruption: Investigations of Long-Term Neurodevelopmental Effects in Rats after Perinatal Exposure to Perfluorohexane Sulfonate (PFHxS). Sci. Rep..

[20] Wang H., Du H., Yang J., Jiang H., Karmin O., Xu L., Liu S., Yi J., Qian X., Chen Y. (2019). PFOS, PFOA, Estrogen Homeostasis, and Birth Size in Chinese Infants. Chemosphere.

[21] European Union (EU) (2025). Regulation (EU) 2025/1988—Amendment to REACH Annex XVII: Restriction of PFAS in Firefighting Foams. http://data.europa.eu/eli/reg/2025/1988/oj.

[22] U.S. Environmental Protection Agency (EPA) (2024). National Primary Drinking Water Regulation for PFAS. https://www.epa.gov/sdwa/and-polyfluoroalkyl-substances-pfas.

[23] Hull S.D., Deen L., Petersen K.U., Jensen T.K., Hammer P., Wils R.S., Frankel H.N., Ostrowski S.R., Tøttenborg S.S. (2023). Time Trends in Per- and Polyfluoroalkyl Substances (PFAS) Concentrations in the Danish Population: A Review Based on Published and Newly Analyzed Data. Environ. Res..

[24] Taucare G., Chan G., Nilsson S., Toms L.-M.L., Zhang X., Mueller J.F., Jolliet O. (2024). Temporal Trends of Per- and Polyfluoroalkyl Substances Concentrations: Insights from Australian Human Biomonitoring 2002–2021 and the U.S. NHANES Programs 2003–2018. Environ. Res..

[25] Figuière R., Miaz L.T., Savvidou E., Cousins I.T. (2025). An Overview of Potential Alternatives for the Multiple Uses of Per- and Polyfluoroalkyl Substances. Environ. Sci. Technol..

[26] Meng L., Zhou B., Liu H., Chen Y., Yuan R., Chen Z., Luo S., Chen H. (2024). Advancing Toxicity Studies of Per- and Poly-Fluoroalkyl Substances (Pfass) through Machine Learning: Models, Mechanisms, and Future Directions. Sci. Total Environ..

[27] Hu J., Yang X., Song X., Liang K., Huang M., Zhao S., Liu H. (2025). Elucidating Environmental Fate and Toxicological Mechanisms of Ultrashort- and Short-Chain PFAS: Integrating Machine Learning, Molecular Modeling, and Experimental Validation. J. Environ. Manag..

[28] Cheng W., Ng C.A. (2019). Using Machine Learning to Classify Bioactivity for 3486 Per- and Polyfluoroalkyl Substances (PFASs) from the OECD List. Environ. Sci. Technol..

[29] Antoniou M., Papavasileiou K.D., Melagraki G., Dondero F., Lynch I., Afantitis A. (2024). Development of a Robust Read-Across Model for the Prediction of Biological Potency of Novel Peroxisome Proliferator-Activated Receptor Delta Agonists. Int. J. Mol. Sci..

[30] Antoniou M., Papavasileiou K.D., Tsoumanis A., Melagraki G., Afantitis A. (2025). Predicting Peroxisome Proliferator-Activated Receptor Gamma Potency of Small Molecules: A Synergistic Consensus Model and Deep Learning Binding Affinity Approach Powered by Enalos Cloud Platform. Mol. Divers..

[31] Bhhatarai B., Gramatica P. (2010). Per- and Polyfluoro Toxicity (LC_50_ Inhalation) Study in Rat and Mouse Using QSAR Modeling. Chem. Res. Toxicol..

[32] Bhhatarai B., Gramatica P. (2011). Oral LD50 Toxicity Modeling and Prediction of Per- and Polyfluorinated Chemicals on Rat and Mouse. Mol. Divers..

[33] Sarkar S., Pore S., Roy K. (2025). A Q-RASAR Approach for Oral and Inhalational Toxicity Prediction of Perfluorinated and Polyfluorinated Compounds (PFCs) Using Rodent Toxicity Data. NAM J..

[34] Feinstein J., Sivaraman G., Picel K., Peters B., Vázquez-Mayagoitia Á., Ramanathan A., MacDonell M., Foster I., Yan E. (2021). Uncertainty-Informed Deep Transfer Learning of Perfluoroalkyl and Polyfluoroalkyl Substance Toxicity. J. Chem. Inf. Model..

[35] Li Y., Fu G.-L., Zhang L., Chen T., Wang Q., Ding C. (2025). Predicting Aquatic Toxicity of Organic Compounds Using the Ml-Dl-Ens Model: An Integrated Approach of Machine Learning and Deep Learning. SSRN.

[36] Bo T., Lin Y., Han J., Hao Z., Liu J. (2023). Machine Learning-Assisted Data Filtering and QSAR Models for Prediction of Chemical Acute Toxicity on Rat and Mouse. J. Hazard. Mater..

[37] Mansouri K., Karmaus A.L., Fitzpatrick J., Patlewicz G., Pradeep P., Alberga D., Alepee N., Allen T.E.H., Allen D., Alves V.M. (2021). CATMoS: Collaborative Acute Toxicity Modeling Suite. Environ. Health Perspect..

[38] U.S. Environmental Protection Agency (EPA) (2022). webTEST: Toxicity Estimation Software Tool, CompTox Chemicals Dashboard. https://comptox.epa.gov/dashboard/predictions.

[39] Cronin M., Madden J., Enoch S., Roberts D. (2013). Chemical Toxicity Prediction.

[40] Benfenati E., Chaudhry Q., Gini G., Dorne J.L. (2019). Integrating in Silico Models and Read-across Methods for Predicting Toxicity of Chemicals: A Step-Wise Strategy. Environ. Int..

[41] Schultz T.W., Cronin M.T.D. (2017). Lessons Learned from Read-across Case Studies for Repeated-Dose Toxicity. Regul. Toxicol. Pharmacol..

[42] Varsou D.-D., Tsiliki G., Nymark P., Kohonen P., Grafström R., Sarimveis H. (2018). toxFlow: A Web-Based Application for Read-Across Toxicity Prediction Using Omics and Physicochemical Data. J. Chem. Inf. Model..

[43] Guo Y., Zhao L., Zhang X., Zhu H. (2019). Using a Hybrid Read-across Method to Evaluate Chemical Toxicity Based on Chemical Structure and Biological Data. Ecotoxicol. Environ. Saf..

[44] Russo D.P., Strickland J., Karmaus A.L., Wang W., Shende S., Hartung T., Aleksunes L.M., Zhu H. (2019). Nonanimal Models for Acute Toxicity Evaluations: Applying Data-Driven Profiling and Read-Across. Environ. Health Perspect..

[45] Drwal M.N., Banerjee P., Dunkel M., Wettig M.R., Preissner R. (2014). ProTox: A Web Server for the in Silico Prediction of Rodent Oral Toxicity. Nucleic Acids Res..

[46] Chen S., Fan T., Zhang N., Zhao L., Zhong R., Sun G. (2024). The Oral Acute Toxicity of Per- and Polyfluoroalkyl Compounds (PFASs) to Rat and Mouse: A Mechanistic Interpretation and Prioritization Analysis of Untested PFASs by QSAR, q-RASAR and Interspecies Modelling Methods. J. Hazard. Mater..

[47] Da Silva N.A.B.R., De Melo E.B. (2024). Analysis of Oral and Inhalation Toxicity of Per- and Polyfluoroalkylated Organic Compounds in Rats and Mice Using Multivariate QSAR. SAR QSAR Environ. Res..

[48] Lu X., Wang X., Chen S., Fan T., Zhao L., Zhong R., Sun G. (2024). The Rat Acute Oral Toxicity of Trifluoromethyl Compounds (TFMs): A Computational Toxicology Study Combining the 2D-QSTR, Read-across and Consensus Modeling Methods. Arch. Toxicol..

[49] Taunk K., De S., Verma S., Swetapadma A. (2019). A Brief Review of Nearest Neighbor Algorithm for Learning and Classification. Proceedings of the 2019 International Conference on Intelligent Computing and Control Systems (ICCS), Madurai, India, 15–17 May 2019.

[50] Breiman L. (2001). Random Forests. Mach. Learn..

[51] Chen T., Guestrin C. (2016). XGBoost: A Scalable Tree Boosting System. Proceedings of the 22nd ACM SIGKDD International Conference on Knowledge Discovery and Data Mining, San Francisco, CA, USA, 13–17 August 2016.

[52] PubChem PubChem. https://pubchem.ncbi.nlm.nih.gov/.

[53] USEPA (2018). Label Review Manual (Chapter 7): Precautionary Statements.

[54] Hong H., Xie Q., Ge W., Qian F., Fang H., Shi L., Su Z., Perkins R., Tong W. (2008). Mold^2^, Molecular Descriptors from 2D Structures for Chemoinformatics and Toxicoinformatics. J. Chem. Inf. Model..

[55] Varsou D.-D., Nikolakopoulos S., Tsoumanis A., Melagraki G., Afantitis A., Mavromoustakos T., Kellici T.F. (2018). Enalos+ KNIME Nodes: New Cheminformatics Tools for Drug Discovery. Rational Drug Design.

[56] Berthold M.R., Cebron N., Dill F., Gabriel T.R., Kötter T., Meinl T., Ohl P., Sieb C., Thiel K., Wiswedel B., Preisach C., Burkhardt H., Schmidt-Thieme L., Decker R. (2008). KNIME: The Konstanz Information Miner. Data Analysis, Machine Learning and Applications.

[57] Kursa M.B., Jankowski A., Rudnicki W.R. (2010). Boruta—A System for Feature Selection. Fundam. Informaticae.

[58] Leach A.R., Gillet V.J. (2007). An Introduction to Chemoinformatics.

[59] OECD (2023). (Q)SAR Assessment Framework: Guidance for the Regulatory Assessment of (Quantitative) Structure Activity Relationship Models and Predictions.

[60] Gissi A., Tcheremenskaia O., Bossa C., Battistelli C.L., Browne P. (2024). The OECD (Q)SAR Assessment Framework: A Tool for Increasing Regulatory Uptake of Computational Approaches. Comput. Toxicol..

[61] Gajewicz A. (2018). How to Judge Whether QSAR/Read-across Predictions Can Be Trusted: A Novel Approach for Establishing a Model’s Applicability Domain. Environ. Sci. Nano.

[62] Zhang S., Golbraikh A., Oloff S., Kohn H., Tropsha A. (2006). A Novel Automated Lazy Learning QSAR (ALL-QSAR) Approach: Method Development, Applications, and Virtual Screening of Chemical Databases Using Validated ALL-QSAR Models. J. Chem. Inf. Model..

[63] Roth A.E., Roth A.E. (1988). Introduction to the Shapley Value. The Shapley Value.

[64] Lundberg S., Lee S.-I. (2017). A Unified Approach to Interpreting Model Predictions. arXiv.

[65] Ponce-Bobadilla A.V., Schmitt V., Maier C.S., Mensing S., Stodtmann S. (2024). Practical Guide to SHAP Analysis: Explaining Supervised Machine Learning Model Predictions in Drug Development. Clin. Transl. Sci..

[66] Todeschini R., Consonni V. (2009). Molecular Descriptors for Chemoinformatics.

[67] Hong H., Slavov S., Ge W., Qian F., Su Z., Fang H., Cheng Y., Perkins R., Shi L., Tong W., Dehmer M., Varmuza K., Bonchev D. (2012). Mold^2^ Molecular Descriptors for QSAR. Statistical Modelling of Molecular Descriptors in QSAR/QSPR.

[68] Chavan S., Friedman R., Nicholls I. (2015). Acute Toxicity-Supported Chronic Toxicity Prediction: A k-Nearest Neighbor Coupled Read-Across Strategy. Int. J. Mol. Sci..

[69] Low Y., Sedykh A., Fourches D., Golbraikh A., Whelan M., Rusyn I., Tropsha A. (2013). Integrative Chemical–Biological Read-Across Approach for Chemical Hazard Classification. Chem. Res. Toxicol..

[70] Sabljić A., Protić M. (1982). Molecular Connectivity: A Novel Method for Prediction of Bioconcentration Factor of Hazardous Chemicals. Chem.-Biol. Interact..

[71] Eddy N.O. (2020). Theoretical Chemistry Study on the Toxicity of Some Polychlorobiphenyl (PCB) Compounds Using Molecular Descriptors. Sci. Afr..

[72] Hall L.H., Kier L.B. (1977). Structure–Activity Studies Using Valence Molecular Connectivity. J. Pharm. Sci..

[73] Jurić A., Gagro M., Nikolić S., Trinajstić N. (1992). Molecular Topological Index: An Application in the QSAR Study of Toxicity of Alcohols. J. Math. Chem..

[74] Lee S., Park K., Ahn H.-S., Kim D. (2010). Importance of Structural Information in Predicting Human Acute Toxicity from in Vitro Cytotoxicity Data. Toxicol. Appl. Pharmacol..

[75] Arthur D.E., Uzairu A., Mamza P., Stephen A.E., Shallangwa G. (2016). Quantum Modelling of the Structure-Activity and Toxicity Relationship Studies of Some Potent Compounds on SR Leukemia Cell Line. Chem. Data Collect..

[76] Jaganathan K., Tayara H., Chong K.T. (2022). An Explainable Supervised Machine Learning Model for Predicting Respiratory Toxicity of Chemicals Using Optimal Molecular Descriptors. Pharmaceutics.

[77] Yang L., Wang Y., Hao W., Chang J., Pan Y., Li J., Wang H. (2020). Modeling Pesticides Toxicity to Sheepshead Minnow Using QSAR. Ecotoxicol. Environ. Saf..

[78] Sun G., Zhang Y., Pei L., Lou Y., Mu Y., Yun J., Li F., Wang Y., Hao Z., Xi S. (2021). Chemometric QSAR Modeling of Acute Oral Toxicity of Polycyclic Aromatic Hydrocarbons (PAHs) to Rat Using Simple 2D Descriptors and Interspecies Toxicity Modeling with Mouse. Ecotoxicol. Environ. Saf..

[79] Mahurkar N.D., Gawhale N.D., Lokhande M.N., Uke S.J., Kodape M.M. (2023). Benzimidazole: A Versatile Scaffold for Drug Discovery and beyond—A Comprehensive Review of Synthetic Approaches and Recent Advancements in Medicinal Chemistry. Results Chem..

[80] Paschke A.-S.K., Brägger Y., Botlik B.B., Staudinger E., Green O., Morandi B. (2025). Carbon-to-Nitrogen Atom Swap Enables Direct Access to Benzimidazoles from Drug-like Indoles. Nat. Chem..

[81] Vitaku E., Smith D.T., Njardarson J.T. (2014). Analysis of the Structural Diversity, Substitution Patterns, and Frequency of Nitrogen Heterocycles among U.S. FDA Approved Pharmaceuticals: Miniperspective. J. Med. Chem..

[82] Wieczorkiewicz P.A., Krygowski T.M., Szatylowicz H. (2024). Substituent Effects and Electron Delocalization in Five-Membered N-Heterocycles. Phys. Chem. Chem. Phys..

[83] Çelik G., Healy S.A., Stolte S., Mayer P., Markiewicz M. (2025). *Daphnia magna* as an Alternative Model for (Simultaneous) Bioaccumulation and Chronic Toxicity Assessment—Controlled Exposure Study Indicates High Hazard of Heterocyclic PAHs. Environ. Sci. Technol..

[84] Cronin M.T.D., Belfield S.J., Briggs K.A., Enoch S.J., Firman J.W., Frericks M., Garrard C., Maccallum P.H., Madden J.C., Pastor M. (2023). Making in Silico Predictive Models for Toxicology FAIR. Regul. Toxicol. Pharmacol..

