# Supplementary material for: Read-Across Structural Analysis of PFAS Acute Oral Toxicity in Rats Powered by the Isalos Analytics Platform’s Automated Machine Learning
Source: Toxics. 2026 Feb 3;14(2):152. doi: 10.3390/toxics14020152 (PMC12944375; doi:10.3390/toxics14020152)
Supplement: Supplementary file 1 [file toxics-14-00152-s001.zip › toxics-4090720 table S2-_QMRF.pdf]

Table S2. Rat PFAS-LD50 kNN/read-across –(Q)SAR model reporting format (QMRF) v.2.1.

	**Element**	**Explanation**
**1.**	**QSAR identifier**	
1.1.	QSAR identifier (title)	Read-across model for the prediction of PFAS LD_{50} values in rats.
1.2	Other related models	1. Chen, S.; Fan, T.; Zhang, N.; Zhao, L.; Zhong, R.; Sun, G. The Oral Acute Toxicity of Per- and Polyfluoroalkyl Compounds (PFASs) to Rat and Mouse: A Mechanistic Interpretation and Prioritization Analysis of Untested PFASs by QSAR, q-RASAR and Interspecies Modelling Methods. J. Hazard. Mater. 2024, 480, 136071. https://doi.org/10.1016/j.jhazmat.2024.136071<br><br>2. Da Silva, N. A. B. R.; De Melo, E. B. Analysis of Oral and Inhalation Toxicity of Per- and Polyfluoroalkylated Organic Compounds in Rats and Mice Using Multivariate QSAR. SAR QSAR Environ. Res. 2024, 35 (10), 877–897. https://doi.org/10.1080/1062936X.2024.2417250<br><br>3. Lu, X.; Wang, X.; Chen, S.; Fan, T.; Zhao, L.; Zhong, R.; Sun, G. The Rat Acute Oral Toxicity of Trifluoromethyl Compounds (TFMs): A Computational Toxicology Study Combining the 2D-QSTR, Read-across and Consensus Modeling Methods. Arch. Toxicol. 2024, 98 (7), 2213–2229. https://doi.org/10.1007/s00204-024-03739-w
1.3.	Software coding the model	Isalos Analytics Platform Beta v2.0.6<br><br>Link to web service: INSIGHT: RatTox
**2.**	**General information**	
2.0	Abstract	A k-Nearest Neighbours (kNN)/read-across model for the prediction of the acute oral toxicity class (high or low) of PFAS in rats. Selected PFAS molecular descriptors are used as input data for the predictions.
2.1.	Date of QMRF	15/12/2025
2.2.	QMRF author(s) and contact details	Aikaterini Theodori (theodori@novamechanics.com)<br>Konstantinos D. Papavasileiou (papavasileiou@novamechanics.com)<br>Andreas Tsoumanis (tsoumanis@novamechanics.com)<br>Georgia Melagraki (georgiamelagraki@gmail.com)<br>Antreas Afantitis (afantitis@novamechanics.com)
2.3.	Date of QMRF update(s)	NA

2.4.	QMRF update(s)	NA
2.5.	Model developer(s) and contact details	Aikaterini Theodori (theodori@novamechanics.com)<br>Konstantinos D. Papavasileiou (papavasileiou@novamechanics.com)<br>Andreas Tsoumanis (tsoumanis@novamechanics.com)<br>Georgia Melagraki (georgiamelagraki@gmail.com)<br>Antreas Afantitis (afantitis@novamechanics.com)
2.6.	Date of model development and/or publication	27/11/2025
2.7.	Reference(s) to main scientific papers and/or software package	
2.8.	Availability of information about the model	The model is proprietary: the source code is confidential; however, the description of the modelling workflow is presented in the original research article, the model is implemented as a public web service, and the curated and enriched dataset used for model development is available in the ChemPharos database.
2.9.	Availability of another QMRF for exactly the same model	NA
**3**	**Defining the endpoint - OECD Principle 1: "A DEFINED ENDPOINT"**	**PRINCIPLE 1: "A DEFINED ENDPOINT". ENDPOINT refers to any physicochemical, biological, or environmental property/activity/effect that can be measured and therefore modelled. The intent of PRINCIPLE 1 (a (Q)SAR should be associated with a defined endpoint) is to ensure clarity in the endpoint being predicted by a given model, since a given endpoint could be determined by different experimental protocols and under different experimental conditions. It is therefore important to identify the experimental system and test conditions that is being modelled by the Q)SAR.**
3.1.	Species	Rat (*Rattus*)
3.2.	Endpoint	Acute oral toxicity LD_{50}.
3.3	Comment on endpoint	The endpoint was classified into two toxicity categories: High and Low toxicity.
3.4.	Endpoint units	mg/kg
3.5.	Dependent variable	For modelling purposes all LD_{50} values were categorised as High or Low toxicity. Based on EPA toxicity categories thresholds, the Low toxicity class includes observations with $LD_{50} > 500$ mg/kg.
3.6.	Experimental protocol	OECD QSAR Assessment:<br>OECD. (Q)SAR Assessment Framework: Guidance for the Regulatory Assessment of (Quantitative) Structure Activity Relationship Models and Predictions; OECD Series on Testing and Assessment; OECD, 2023.

		https://doi.org/10.1787/d96118f6-en
3.7.	Endpoint data quality and variability	Dataset class distributions<br>*Subset* — *Low toxicity* — *High toxicity*<br>*Training* — 36% — 64%<br>*Testing* — 37% — 63%
4	**Defining the algorithm - OECD Principle 2 : "AN UNAMBIGUOUS ALGORITHM"**	**PRINCIPLE 2: "AN UNAMBIGUOUS ALGORITHM". The (Q)SAR estimate of an endpoint is the result of applying an ALGORITHM to a set of structural parameters which describe the chemical structure. The intent of PRINCIPLE 2 (a (Q)SAR should be associated with an unambiguous algorithm) is to ensure transparency in the model algorithm that generates predictions of an endpoint from information on chemical structure and/or physicochemical properties. In this context, algorithm refers to any mathematical equation, decision rule or output approach.**
4.1.	Type of model	Instance based/read-across, k-Nearest Neighbours (kNN).
4.2.	Explicit algorithm	The *k*NN/read-across model employs the *k*-nearest neighbours approach, an instance-based method that predicts the endpoint of a compound based on its *k* nearest neighbours in the data space. The proximity between compounds is measured using Euclidean distance, which is adjusted slightly for categorical descriptor values using a binary value (0 in the case of same class data points or 1, otherwise). The endpoint prediction, in this case the toxicity class, is the weighted average of the endpoint values of the *k* closest neighbours (*k* = 3), with each neighbour's weighting factor inversely proportional to its distance from the evaluated compound.
4.3.	Descriptors in the model	*Molecular descriptor* — *Explanation*<br>*D209* — Average vertex connectivity order-3 index.<br>*D207* — Average vertex connectivity order-1 index.<br>*D223* — Average valence vertex connectivity order-5 index.<br>*D488* — Moran topological structure autocorrelation length-2 weighted by atomic van der Waals volumes.<br>*D484* — Moran topological structure autocorrelation length-6 weighted by atomic masses.<br>*D144* — Mean atomic van der Waals carbon-scale.

4.4.	Descriptor selection	Feature selection was implemented using the Boruta method in Isalos Analytics Platform.
4.5.	Algorithm and descriptor generation	Mold2: Mold2 is a software developed by the National Center for Toxicological Research (NCTR) that calculates a large and diverse set of 777 molecular descriptors encoding two-dimensional chemical structure information. https://www.fda.gov/science-research/bioinformatics-tools/mold2
4.6.	Software name and version for descriptor generation	Enalos+ KNIME nodes: "EnalosMold2" node that uses the Mold2.exe executable and an SDF file to calculate molecular descriptors.
4.7.	Chemicals/Descriptors ratio	211 chemicals/ 6 descriptors
**5**	**Defining the applicability domain - OECD Principle 3: "A DEFINED DOMAIN OF APPLICABILITY"**	**PRINCIPLE 3: "A DEFINED DOMAIN OF APPLICABILITY". APPLICABILITY DOMAIN refers to the response and chemical structure space in which the model makes predictions with a given reliability. Ideally the applicability domain should express the structural, physicochemical and response space of the model. The CHEMICAL STRUCTURE (x variable) space can be expressed by information on physicochemical properties and/or structural fragments. The RESPONSE (y variable) can be any physicochemical, biological or environmental effect that is being predicted. According to PRINCIPLE 3 a (Q)SAR should be associated with a defined domain of applicability. Section 5 can be repeated (e.g., 5.a, 5.b, 5.c, etc) as many times as necessary if more than one method has been used to assess the applicability domain.**
5.1.	Description of the applicability domain of the model	The applicability domain is defined by fixed boundaries, the APD threshold (§5.2), calculated by considering Euclidean distances between all molecules in the training set. The distance of a test compound to its nearest neighbour in the training set is compared to the predefined applicability domain threshold. If the distance is beyond this threshold, then the prediction is considered unreliable.
5.2.	Method used to assess the applicability domain	The distance of a test molecule to its nearest neighbour in the training set in compared to the pre-defined APD threshold, APD=<d>+stdev*z. First, the average Euclidean distance between all pairs of training data is calculated and then the set of distances that were lower than the average is formulated. The <d> and stdev values are finally determined as the average and standard deviation of all distances included in the remaining set. z is an empirical parameter with a value of 0.5.
5.3.	Software name and version for applicability domain assessment	Isalos Analytics Platform: "APD" function.

<table>
<tr><td>5.4.</td><td>Limits of applicability</td><td>APD threshold = 2.149</td></tr>
<tr><td>6</td><td>Defining goodness-of-fit and robustness (internal validation) – OECD Principle 4: “APPROPRIATE MEASURES OF GOODNESS-OF-FIT, ROBUSTENESS AND PREDICTIVITY”</td><td>PRINCIPLE 4: “APPROPRIATE MEASURES OF GOODNESS-OF-FIT, ROBUSTENESS AND PREDICTIVITY”. PRINCIPLE 4 expresses the need to perform validation to establish the performance of the model. GOODNESS-OF-FIT and ROBUSTNESS refer to the internal model performance.</td></tr>
<tr><td>6.1.</td><td>Availability of the training set</td><td>The training set is available in the ChemPharos database: ChemPharos Dataset Query Page</td></tr>
<tr><td>6.2.</td><td>Available information for the training set</td><td>a) Chemical names (IUPAC names); b) CAS numbers; c) SMILES; d) MW; e) LD50 mg/kg; f) LD50 mol/kg; g) -log(LD50).</td></tr>
<tr><td>6.3.</td><td>Data for each descriptor variable for the training set</td><td>The training set is available in the ChemPharos database: ChemPharos Dataset Query Page</td></tr>
<tr><td>6.4.</td><td>Data for the dependent variable for the training set</td><td>The training set is available in the ChemPharos database: ChemPharos Dataset Query Page</td></tr>
<tr><td>6.5.</td><td>Other information about the training set</td><td>Stratified random sampling (70/30 ratio) was used for data partitioning, retaining the original class ratio in the training and testing sets: 211 out of 307 PFAS were included in the training set for model development. The ratio of Low to High class observations is mentioned in §3.7.</td></tr>
<tr><td>6.6.</td><td>Pre-processing of data before modelling</td><td>Removal of columns with repeated values (cutoff limit of 20% to filter out columns), Gaussian normalization of descriptors (z-score). Variable selection was performed according to §4.4.</td></tr>
<tr><td>6.7.</td><td>Statistics for goodness-of-fit</td><td>Training set:
<table>
<tr><th>Accuracy</th><th>Precision</th><th>Sensitivity</th><th>Specificity</th><th>F1-score</th><th>MCC</th></tr>
<tr><td>1.0</td><td>1.0</td><td>1.0</td><td>1.0</td><td>1.0</td><td>1.0</td></tr>
</table></td></tr>
<tr><td>6.8.</td><td>Robustness - Statistics obtained by leave-one-out cross-validation</td><td>NA</td></tr>
<tr><td>6.9.</td><td>Robustness - Statistics obtained by leave-many-out cross-validation</td><td>10-fold cross-validation on the training set:
<table>
<tr><th>Metric</th><th>Accuracy</th><th>Precision</th><th>Sensitivity</th><th>Specificity</th><th>F1-score</th><th>MCC</th></tr>
<tr><td>Value</td><td>0.829</td><td>0.773</td><td>0.824</td><td>0.811</td><td>0.794</td><td>0.623</td></tr>
</table></td></tr>
<tr><td>6.10.</td><td>Robustness - Statistics obtained by Y-scrambling</td><td>10 iterations: Them mean accuracy and MCC values are reported alongside their standard deviations (σ).
<table>
<tr><th>Metric</th><th>Accuracy</th><th>MCC</th></tr>
<tr><td>Value ± σ</td><td>0.568 ±0.039</td><td>0.041 ±0.097</td></tr>
</table></td></tr>
<tr><td>6.11.</td><td>Robustness - Statistics obtained by bootstrap</td><td>NA</td></tr>
<tr><td>6.12.</td><td>Robustness - Statistics obtained by other methods</td><td>NA</td></tr>
</table>

<table>
<tr><td>7</td><td>Defining predictivity (external validation) – OECD Principle 4: “APPROPRIATE MEASURES OF GOODNESS-OF-FIT, ROBUSTENESS AND PREDICTIVITY”</td><td>PRINCIPLE 4: “APPROPRIATE MEASURES OF GOODNESS-OF-FIT, ROBUSTENESS AND PREDICTIVITY”. PRINCIPLE 4 expresses the need to perform validation to establish the performance of the model. PREDICTIVITY refers to the external model validation. Section 7 can be repeated (e.g., 7.a, 7.b, 7.c, etc) as many times as necessary if more validation studies need to be reported in the QMRF.</td></tr>
<tr><td>7.1.</td><td>Availability of the external validation set</td><td>The external validation (testing) set is available in the ChemPharos database:
ChemPharos Dataset Query Page</td></tr>
<tr><td>7.2.</td><td>Available information for the external validation set</td><td>a) Chemical names (IUPAC names); b) CAS numbers; c) SMILES; d) MW; e) LD50 mg/kg; f) LD50 mol/kg; g) -log(LD50).</td></tr>
<tr><td>7.3.</td><td>Data for each descriptor variable for the external validation set</td><td>The external validation (testing) set is available in the ChemPharos database:
ChemPharos Dataset Query Page</td></tr>
<tr><td>7.4.</td><td>Data for the dependent variable for the external validation set</td><td>The external validation (testing) set is available in the ChemPharos database:
ChemPharos Dataset Query Page</td></tr>
<tr><td>7.5.</td><td>Other information about the external validation set</td><td>92 PFAS were included in the testing set, which was not involved in model development, but it was rather used solely for validating purposes.</td></tr>
<tr><td>7.6.</td><td>Experimental design of test set</td><td>The testing set was selected using a stratified random partitioning.</td></tr>
<tr><td>7.7.</td><td>Predictivity - Statistics obtained by external validation</td><td><table><tr><th>Metric</th><th>Accuracy</th><th>Precision</th><th>Sensitivity</th><th>Specificity</th><th>F1-score</th><th>MCC</th></tr><tr><td>Value</td><td>0.815</td><td>0.758</td><td>0.735</td><td>0.862</td><td>0.746</td><td>0.601</td></tr></table></td></tr>
<tr><td>7.8.</td><td>Predictivity - Assessment of the external validation set</td><td>The external testing set is 30% of the initial dataset, 98.9% of predictions fall within the domain of applicability (threshold = 2.149).</td></tr>
<tr><td>7.9.</td><td>Comments on the external validation of the model</td><td>The testing set was normalized based on the Gaussian normalization applied on the training set.</td></tr>
<tr><td>8</td><td>Providing a mechanistic interpretation - OECD Principle 5: “A MECHANISTIC INTERPRETATION, IF POSSIBLE”</td><td>PRINCIPLE 5: “A MECHANISTIC INTERPRETATION, IF POSSIBLE”. According to PRINCIPLE 5, a (Q)SAR should be associated with a mechanistic interpretation, if possible.</td></tr>
<tr><td>8.1.</td><td>Mechanistic basis of the model</td><td>The SHAP analysis indicates that bulky, densely packed structures at short atomic ranges are potential markers of high toxicity, whereas a more balanced mass and electron density distribution at larger atomic ranges shows the opposite association. Furthermore, read-across structural analysis of representative testing-set PFAS identified polyaromatic or heterocyclic moieties in the PFAS structure</td></tr>
</table>

		as contributors to higher toxicity, in contrast to simpler linear PFAS molecules lacking heteroatoms.
8.2.	A priori or a posteriori mechanistic interpretation	A posteriori (after modelling, by interpretation of the final set of training structures and or descriptors).
8.3.	Other information about the mechanistic interpretation	NA
**9**	**Miscellaneous information**	
9.1.	Comments	Model development was performed within the Isalos autoML scheme. In this report only the final model is documented. The final model was selected as the kNN model achieving the highest accuracy in 5-fold cross validation. Once the optimal hyperparameters were identified for the algorithm, the model was re-trained using the full training set. Its generalisability was then assessed on the testing set comprised of 92 PFAS observations from the original set.
9.2.	Bibliography	1. The original data were retrieved from the studies of Chen, S.; Fan, T.; Zhang, N.; Zhao, L.; Zhong, R.; Sun, G. The Oral Acute Toxicity of Per- and Polyfluoroalkyl Compounds (PFASs) to Rat and Mouse: A Mechanistic Interpretation and Prioritization Analysis of Untested PFASs by QSAR, q-RASAR and Interspecies Modelling Methods. J. Hazard. Mater. 2024, 480, 136071. https://doi.org/10.1016/j.jhazmat.2024.136071 , Da Silva, N. A. B. R.; De Melo, E. B. Analysis of Oral and Inhalation Toxicity of Per- and Polyfluoroalkylated Organic Compounds in Rats and Mice Using Multivariate QSAR. SAR QSAR Environ. Res. 2024, 35 (10), 877–897. https://doi.org/10.1080/1062936X.2024.2417250 and Lu, X.; Wang, X.; Chen, S.; Fan, T.; Zhao, L.; Zhong, R.; Sun, G. The Rat Acute Oral Toxicity of Trifluoromethyl Compounds (TFMs): A Computational Toxicology Study Combining the 2D-QSTR, Read-across and Consensus Modeling Methods. Arch. Toxicol. 2024, 98 (7), 2213–2229. https://doi.org/10.1007/s00204-024-03739-w<br>2. Read more about the Mold2 descriptors: Hong, H.; Xie, Q.; Ge, W.; Qian, F.; Fang, H.; Shi, L.; Su, Z.; Perkins, R.; Tong, W. Mold2, Molecular Descriptors from 2D Structures for Chemoinformatics and Toxicoinformatics. J. Chem. Inf. Model. 2008, 48 (7),

		1337–1344. https://doi.org/10.1021/ci800038f<br>3. Model development and dissemination were performed in Isalos Analytics Platform: Introduction | Isalos Analytics Platform Docs
9.3	Supporting information	The curated and enriched dataset is available in the ChemPharos database: ChemPharos Dataset Query Page
