# Supplementary material for: Read-Across Structural Analysis of PFAS Acute Oral Toxicity in Rats Powered by the Isalos Analytics Platform’s Automated Machine Learning
Source: Toxics. 2026 Feb 3;14(2):152. doi: 10.3390/toxics14020152 (PMC12944375; doi:10.3390/toxics14020152)
Supplement: Supplementary file 1 [file toxics-14-00152-s001.zip › toxics-4090720-table S1 and Figure S1.pdf]

Read-across structural analysis of PFAS acute oral toxicity in rats powered by the Isalos Analytics Platform's Automated Machine Learning

Aikaterini Theodori[1], Konstantinos D. Papavasileiou[1,2], Andreas Tsoumanis[1,2,3], Georgia Melagraki[4], Antreas Afantitis[1,2,3,5,*]

[1]Department of ChemInformatics, NovaMechanics MIKE, 18545 Piraeus, Greece
[2]Department of ChemInformatics, NovaMechanics Ltd, 1070 Nicosia, Cyprus
[3]Entelos Institute, 6059 Larnaca, State, Cyprus
[4]Division of Physical Sciences Applications, Hellenic Military Academy, 16672 Vari, Greece
[5]Department of Pharmacy, Frederick University, Nicosia 1036, Cyprus
* Correspondence: author(s). E-mail(s): afantitis@novamechanics.com

1. *Descriptor set evaluation and selection*

The development of robust and reliable QSAR models depends, among other factors, on the selection of an appropriate molecular descriptor set for each application. In this study, we systematically evaluated two distinct molecular descriptor calculation methods: Mold2 descriptors and Morgan fingerprints. The PFAS SMILES representations were converted into SDF format and processed using the *Enalos Mold2* and *RDKit Fingerprints* nodes within the KNIME Analytics Platform. The same randomised, stratified training and testing split described in the main publication was maintained for both descriptor sets to enable a fair comparative analysis.

The *RDKit Fingerprints* node was configured to generate Morgan fingerprints of 1024 bits with a radius of 2. The resulting fingerprint vectors were expanded into 1024 individual columns and used directly for model training, without prior variable selection or normalisation. Consequently, the Isalos AutoML function was employed for model hyperparameter tuning via 5-fold cross-validation in the data subspace defined in Table 1 of the main publication, using the same AutoML random seed (seed = 1764664892465). The achieved 5-fold accuracy metric along with the optimal hyperparameter values selected for the kNN, XGBoost, RF, and NN models trained on the Morgan fingerprints set are presented in Table S1.

Table S1. AutoML optimisation results of a kNN, XGBoost, RF, and NN model trained on the Morgan fingerprints descriptor set. The optimal hyperparameters are reported along with the maximised average accuracy statistic achieved during cross-validation.

ML method	*Hyperparameter*	*Optimal value*	*5-fold accuracy*
kNN	Number of nearest neighbours, k	4	0.819
XGBoost	Number of trees	20	0.786
	Learning rate	0.3	
	Gamma	0	
	Max tree depth	6	
	Minimum child weight	1	

	Column sample by tree	1	
	Subsample	1	
	Lambda	1	
	Alpha	1	
Random Forest	Features fracture	0.5	0.800
	Min impurity decrease	0	
	Number of ensembles	150	
Fully Connected Neural Network	Number of Hidden Layers	2	0.800
	Number of Neurons/Layer	50	
	Activation Function	RELU	
	Batch Size	128	
	Number of Epochs	150	
	Learning Rate	0.01	
	Momentum	0.9	

The maximum 5-fold cross-validation accuracy achieved across all models using the Morgan fingerprints datasets was consistently lower than that obtained with the Mold2 descriptors (Table 4, main publication). As a result, Mold2 descriptors were deemed more suitable for this application and were subsequently used to develop and evaluate the best-performing model. In addition, the selected descriptor set allows for mechanistic interpretability and enhanced model explainability compared to the vector-based Morgan fingerprints, which is essential for gaining a deeper understanding of the QSAR model.

2. *Pearson's correlation test on the training set*

Although correlation-based filtering was not applied during data preprocessing, the final Mold2 descriptor set was evaluated for inter-correlation using the Pearson correlation coefficient (calculated in Python using the pandas library) to mitigate the risk of overfitting. As shown in the correlation heatmap (Figure S1) no meaningful correlations between the six Mold2 descriptors selected for model development were detected (correlation coefficients < 0.8), ensuring robustness of the model input.

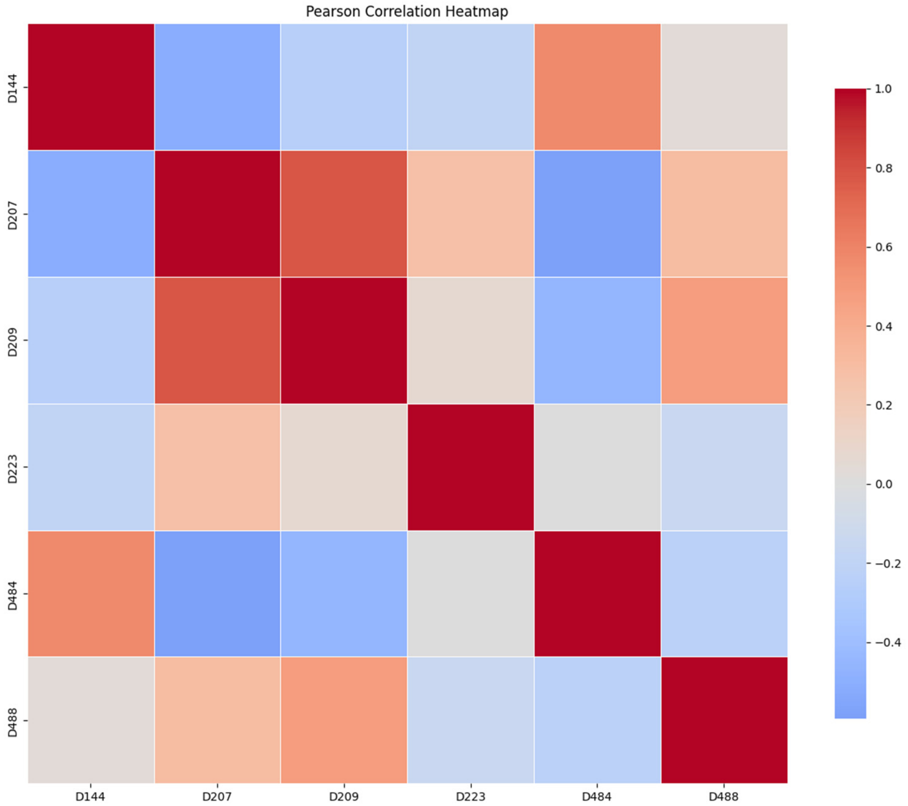

Figure S1. Pearson correlation heatmap of the selected input Mold2 descriptors.
